# A noncanonical function of the tyrosine degradation enzyme FAH drives CDK4/6 inhibitor resistance in breast cancer

**DOI:** 10.1126/sciadv.aef1145

**Published:** 2026-09-30

**Authors:** Jenny Högström, Hanna M. Doh, Aidan Kump, Boryana Petrova, Peng Wang, Jonathan D. Lee, Kaisa Koivula, Nina Kozlova, Kayla A. Cruz, Su Min Hong, Camila Perea, Arjun Garg, Michal Weitman, Conor L. Evans, Jonathan L. Coloff, Jaymin M. Patel, Gerburg M. Wulf, Laura C. Collins, Naama Kanarek, Taru Muranen

**Affiliations:** ^1^ Cancer Research Institute, Beth Israel Deaconess Medical Center, Harvard Medical School, Boston, MA, USA.; ^2^ Department of Pathology, Boston Children’s Hospital, Harvard Medical School, Boston, MA, USA.; ^3^ Core Facilities, Medical University of Vienna, Vienna, Austria.; ^4^ Wellman Center for Photomedicine, Massachusetts General Hospital, Harvard Medical School, Boston, MA, USA.; ^5^ Department of Physiology and Biophysics, University of Illinois Cancer Center, University of Illinois College of Medicine, Chicago, IL, USA.; ^6^ Department of Pathology, and Laboratory Medicine, Weill Cornell Medicine, New York Presbyterian Hospital, New York, NY, USA.

## Abstract

Resistance to standard-of-care therapies remains a major clinical challenge in the treatment of the most common breast cancer, the hormone receptor–positive (HR+) subtype. Cyclin-dependent kinase 4/6 (CDK4/6) inhibitors improve outcomes in early-stage HR+ disease, yet many patients relapse. Resistance mechanisms of relapsed tumors include genetic alterations, but in many cases, no genetic drivers are identified. Here, we investigated mechanisms underlying resistance to CDK4/6 inhibitors using breast cancer patient–derived models and tumors. We identified an unexpected, noncanonical nuclear function of fumarylacetoacetate hydrolase (FAH), an enzyme in the tyrosine catabolism pathway, as a driver of resistance. FAH translocated to the nucleus upon CDK4/6 inhibition, where it interacted with cyclin-dependent kinase 9 (CDK9), and promoted resistance. Nuclear FAH was enriched in tumors from relapsed patients, and inhibition of CDK9 reversed FAH-mediated resistance. These findings establish nuclear FAH as a biomarker of resistance and revealed CDK9 as a therapeutic vulnerability in CDK4/6 inhibitor–resistant HR+ breast cancer.

## INTRODUCTION

Breast cancer is the most common cancer in women with 2.3 million annual cases globally (*1*). Hormone receptor positive (HR+) breast cancer is the most common subtype, accounting for ∼70% of newly diagnosed breast cancer cases. Early-stage HR+ breast cancers are treated with endocrine therapies (ETs) such as tamoxifen and aromatase inhibitors (*2*). Although early-stage HR+ breast cancers usually respond well to treatment and can be cured, ∼30% of the patients relapse (*3*). Relapsed tumors have usually metastasized and acquired resistance to ETs (*4*). Recurrent HR+ breast cancers are treated with ET in combination with additional targeted therapies, including CDK4/6 inhibitors (*5*–*7*). CDK4/6 inhibitors synergize with ET to induce cell cycle arrest and senescence. CDK4/6 inhibitors have become the standard of care for metastatic HR+ breast cancer and have improved the outcome for both high-risk early-stage breast cancers and metastatic breast cancers (*8*–*10*). Unfortunately, ∼20% of patients show de novo resistance to ET and CDK4/6 inhibitor therapy, and most of the patients that initially respond develop adaptive resistance (*11*). Recent efforts have identified several putative genetic drivers of resistance for CDK4/6 inhibitors, such as *RB* loss, *CCNE2*, *FGFR2*, and *AURKA* amplification (*12*–*14*). However, most of the patients do not harbor these alterations, and so these genetic alterations do not distinguish responders from nonresponders and thereby underscore the need for discovery of yet unknown resistance mechanisms and predictive biomarkers.

Cancer cells in poorly vascularized microenvironments use alternative sources of nutrients to maintain the synthesis of biomolecules, energy demand, and other metabolic needs, such as redox homeostasis (*15*). Deprivation of essential nutrients causes reprogramming of anabolic and catabolic pathways, such as glucose, fatty acid, and amino acid metabolism, ultimately enabling tumor growth, disease progression, and therapy resistance (*16*). Tumor growth is fueled by amino acids, which provide most of the carbon-based mass for rapidly proliferating cells (*17*). In addition to contributing to the carbon pool, amino acid catabolism provides building blocks for nucleotides, lipids, redox homeostasis, and epigenetic regulation (*18*). Altered amino acid metabolism, such as methionine, glutamine, and serine metabolism, is frequently observed in breast cancer where it fuels tumor growth (*19*). In addition to tumor growth, enhanced amino acid metabolism can contribute to resistance to chemotherapy and targeted therapies, such as ET in breast cancer (*20*–*22*). However, many amino acid pathways intersect, and amino acid enzymes can perform functions outside of amino acid metabolism; therefore, their function in disease progression and drug resistance is complex and sometimes unknown.

In this study, we sought to analyze enhanced amino acid pathways in breast cancer to identify metabolic vulnerabilities that drive disease progression and treatment resistance. In addition to previously known amino acid pathways, we observed that tyrosine metabolism pathway is enriched in breast cancer and that fumarylacetoacetate hydrolase (FAH), the last enzyme in the tyrosine degradation pathway, is overexpressed in HR+ breast cancer where it stimulates resistance to CDK4/6 inhibitors. However, despite its canonical function in tyrosine catabolism, *FAH*-overexpressing cells do not use the tyrosine catabolism pathway. Unexpectedly, we found that FAH translocates to the nucleus during CDK4/6 inhibition, as observed in patient-derived organoids (PDOs) and in patient samples, and that the nuclear localization is essential for FAH-mediated resistance and independent of the tyrosine catabolism pathway. Mechanistically, we show that FAH interacts with CDK9 to drive CDK4/6 inhibitor resistance. Collectively, this suggests a yet uncharacterized moonlighting function of FAH in the nucleus and links this metabolic enzyme to treatment resistance in HR+ breast cancer.

## RESULTS

### FAH is enriched in HR+ breast cancer and correlates with poor disease outcome

Given the major contribution of aberrant metabolism to the growth and therapy resistance in breast cancer, we explored altered amino acid metabolism pathways in breast cancer. We performed an in silico analysis on The Cancer Genome Atlas (TCGA) Pan Cancer Atlas dataset to compare amino acid pathways in normal breast tissue and breast cancer (*23*). We observed that multiple amino acid metabolism pathways were enriched in breast tumors (Fig. 1A), including tyrosine metabolism that has been shown to affect cell growth in amino acid starved triple negative breast cancer cells (*24*). Our analysis revealed that tyrosine aminotransferase (*TAT*) and *FAH*, the first and last enzymes in the tyrosine degradation pathway, respectively, were enriched in breast tumors (Fig. 1B and fig. S1A). To test the correlation between these genes and disease outcome, we assessed patient overall survival in high and low *TAT* and *FAH* mRNA expressing tumors and observed that high expression of both *FAH* and *TAT* correlated with poor overall outcome (fig. S1, B and C). Since FAH functions downstream of the irreversible and committed step in the canonical tyrosine degradation pathway, we further analyzed its expression and function in different subtypes of breast cancer. We observed that *FAH* mRNA is particularity high in HR+ and HER2-amplified breast cancer (Fig. 1C) and correlates with both poor overall and disease-free survival in HR+ breast cancer (Fig. 1, D and E). To assess whether we can detect enrichment of FAH protein in breast tumors, we stained a tissue microarray (TMA) consisting of multiple cores of normal breast tissue and breast tumors of different subtypes (data S1). Similar to mRNA levels, we observed a significant enrichment of FAH protein in HR+ breast tumors (Fig. 1, F and G), and a clinical pathology scoring of these stained samples confirmed the highest score for HR+ tumors (Fig. 1, G and H). Furthermore, although we detected variable FAH expression levels in multiple breast cancer cell lines of different subtypes, FAH was highly expressed in most of the ER+ and HER+ cell lines (Fig. 1I). Collectively, these data suggest that FAH may have a function in HR+ breast cancer.

**Fig. 1.**
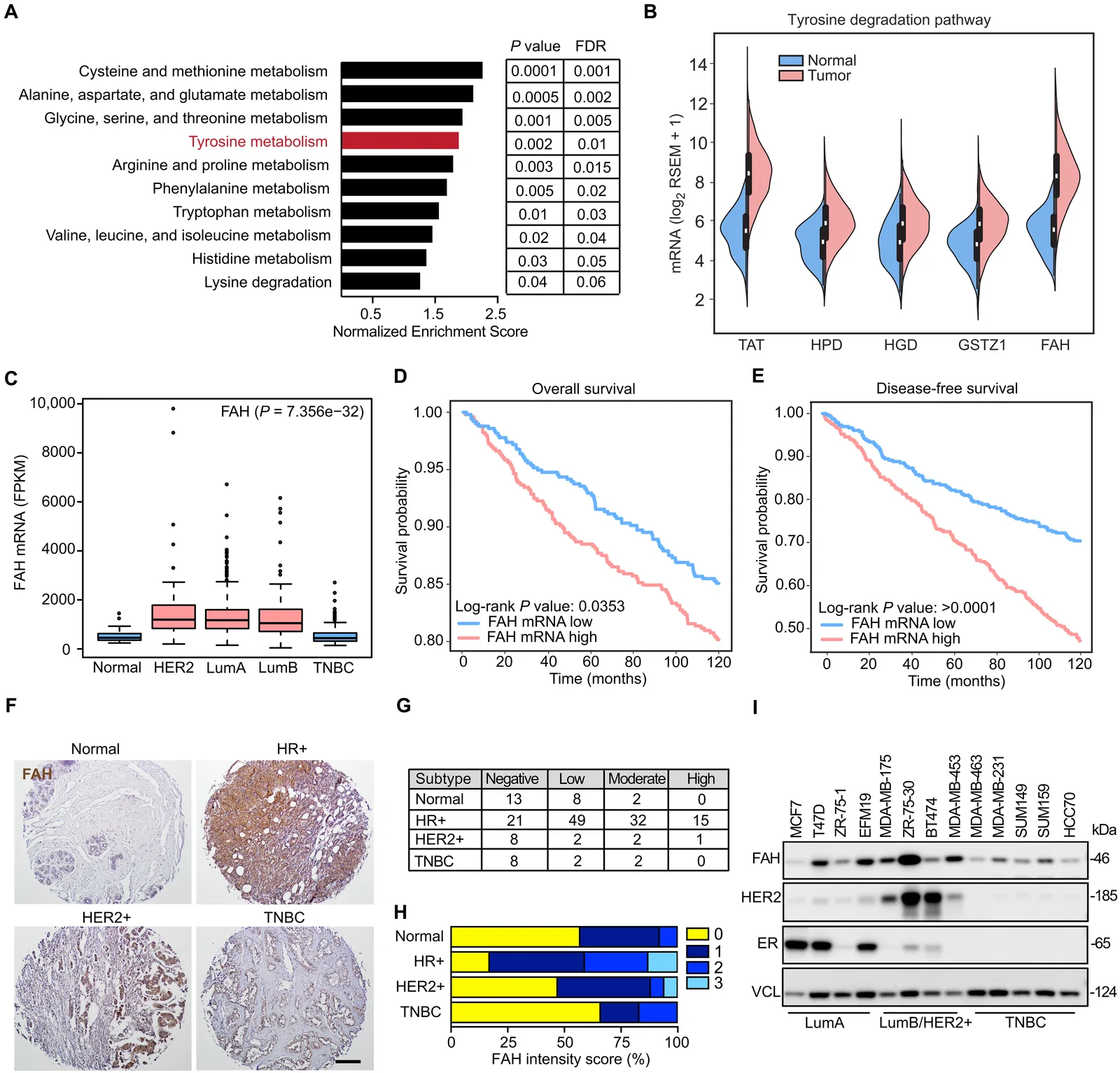
FAH is enriched in HR+ breast cancer and correlates with poor survival. (**A**) Gene set enrichment analysis of amino acid metabolism pathways in normal breast tissue and breast cancer. The KEGG pathway database was used for the enrichment analysis. The data are presented as normalized enrichment scores, *P* value, and false discovery rate (FDR) for each metabolic pathway. (**B**) Tyrosine catabolism enzymes *TAT*, *HPD*, *homogentisate 1,2-dioxygenase* (*HGD*), *glutathione *S*-transferase zeta 1* (*GSTZ1*), and *FAH* mRNA in normal breast tissue and breast cancer. White dots represent the mean expression. (**C**) *FAH* mRNA levels in different breast cancer subtypes [triple negative breast cancer (TNBC)]. (**D** and **E**) Kaplan-Meyer overall survival (D) and disease-free survival (E) plot of *FAH* high versus low mRNA in HR+ breast cancer. A tertile-based cutoff was used to determine *FAH* high and low mRNA. (**F** to **H**) Representative images of FAH immunohistochemistry (F) and scoring [(G) and (H)] of normal breast tissue and breast tumors of different subtypes from a TMA containing 192 cores representing 189 total cases with 21 adjacent normal tissue, 4 cancer adjacent tissue, and 7 normal tissue cores. FAH staining scores: 0 = negative, 1 = low, 2 = moderate, 3 = high. (**I**) FAH, HER2, estrogen receptor alpha (ER), and vinculin (VCL) proteins were probed by Western blot in indicated breast cancer cell lines. The TCGA Pan-Cancer Atlas [(A), (B), and (D)] or the TCGA Firehose legacy (C) datasets were used for the analysis. Two-sample *t* test (A), two-sample *t* test with Bonferroni correction (B), analysis of variance (ANOVA) (C), or Mantel-Cox log rank test (D) was used to assess significance. Scale bar, 200 μm.

### FAH stimulates resistance to CDK4/6 inhibitors

Because we observed that FAH was enriched in HR+ breast cancer and correlated with poor disease outcome, we next asked whether FAH promotes tumor growth and/or resistance to targeted therapies used in HR+ breast cancer management. To assess this, we overexpressed *FAH* in MCF7 cells that have low FAH expression (Fig. 1I). Although *FAH* overexpression did not change cell proliferation in vehicle-treated cells (fig. S2A), we observed a significant increase in the abemaciclib median inhibitory concentration (IC_50_) and percentage of proliferating cells [measured by 5-ethynyl-2′-deoxyuridine–positive (EdU+) pulsing] in *FAH*-overexpressing cells (Fig. 2, A and B, and fig. S2A). Conversely, *FAH* knockout in the cell lines T47D and MDA-MB-175 that express high FAH levels (Fig. 1I) reduced the abemaciclib IC_50_ (Fig. 2C and fig. S2, B and C). To validate this in a more clinically relevant model system, we stained a panel of HR+ PDOs for FAH and observed high cytoplasmic and, unexpectedly, nuclear expression of FAH in two of the four HR+ PDOs (patient #8 and patient #10) which were derived from patients relapsed on CDK4/6 inhibitors (Fig. 2D and data S1). We next assessed whether *FAH* knockout in the two FAH-high expressing PDOs would sensitize them to CDK4/6 inhibitors abemaciclib and palbociclib. Similar to our data with breast cancer cell lines, *FAH*-KO PDOs showed increased sensitivity to CDK4/6 inhibition (Fig. 2, E to I, and fig. S2, D to G). Next, we overexpressed *FAH* in the FAH-negative and the FAH-low PDO lines (patient #26 and patient #27) and observed that the *FAH*-overexpressing PDOs were more resistant to CDK4/6 inhibitors (Fig. 2, J to N, and fig. S2, H and I), showing that FAH increases resistance to CDK4/6 inhibitors.

**Fig. 2.**
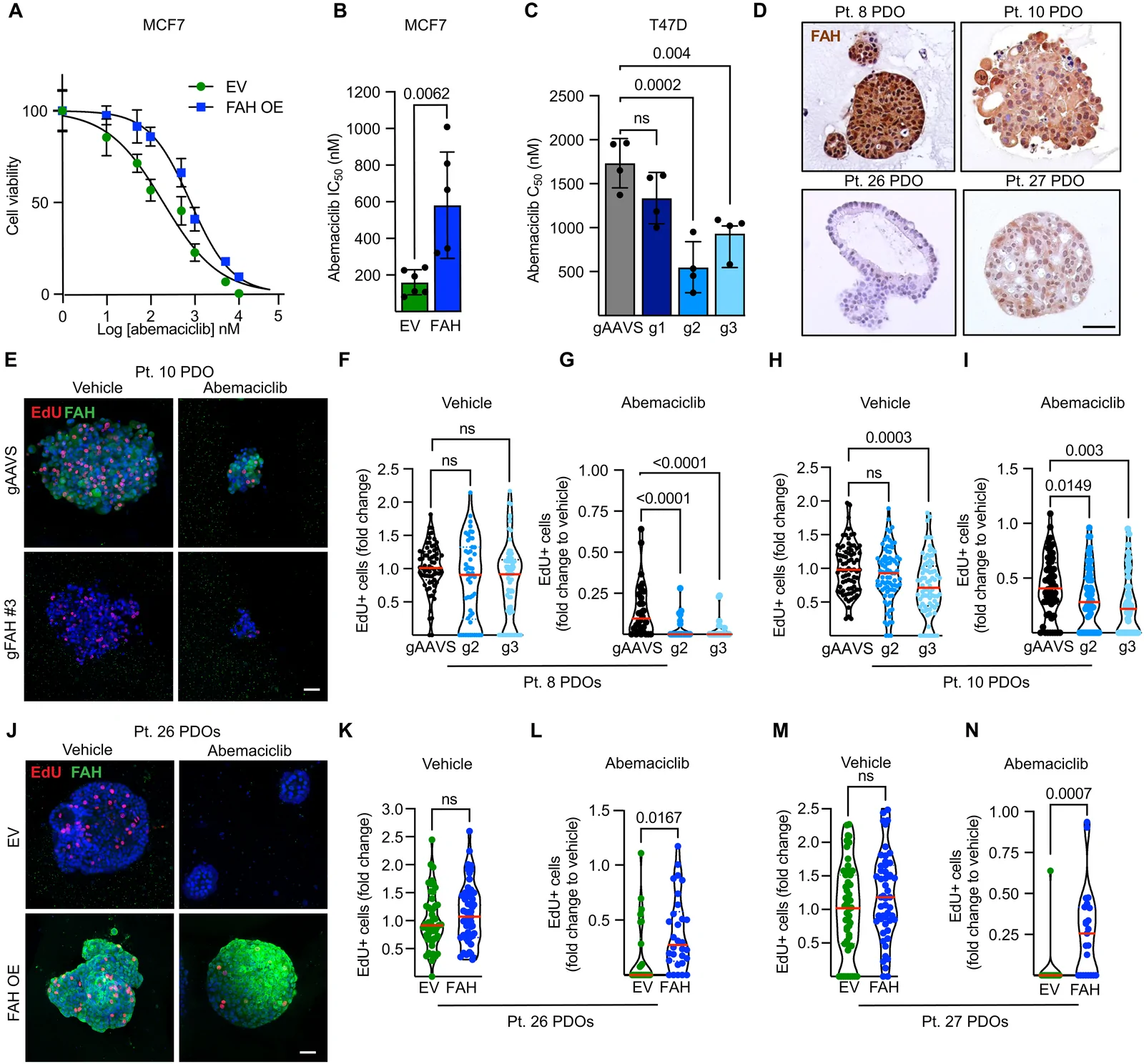
FAH stimulates resistance to CDK4/6 inhibitors. (**A** and **B**) IC_50_ curve (A) and IC_50_ value (B) of empty vector (EV)– and *FAH*-overexpressing MCF7 cells treated with abemaciclib for 72 hours. (**C**) IC_50_ value in T47D gAAVS and gFAH cells treated with abemaciclib for 72 hours. Three guide RNAs (#g1-3) were used to knock out *FAH* by CRISPR-Cas9. (**D**) Representative images of FAH immunohistochemistry in patients #8, #10, #26, and #27 PDOs. (**E**) Representative images of EdU and FAH staining in patient #10 gAAVS and gFAH#3 PDOs treated with vehicle or 300 nM abemaciclib. (**F** and **G**) Quantification of EdU+ cells in patient #8 gAAVS and gFAH PDOs treated with vehicle (F) or 300 nM abemaciclib (G). (**H** and **I**) Quantification of EdU+ cells in patient #10 gAAV and gFAH PDOs treated with vehicle (H) or 300 nM abemaciclib (I). (**J**) Representative images of EdU and FAH staining in patient #26 EV- and FAH-overexpressing PDOs treated with vehicle or 300 nM abemaciclib. (**K** and **L**) Quantification of EdU+ cells in patient #26 EV- and *FAH*-overexpressing PDOs treated with vehicle (K) or 300 nM abemaciclib (L). (**M** and **N**) Quantification of EdU+ cells in patient #27 EV- and *FAH*-overexpressing PDOs treated with vehicle (M) or 300 nM abemaciclib (N). PDOs were treated with 300 nM abemaciclib for 7 days, whereafter proliferation was assessed by pulsing PDOs with EdU for 4 hours and quantifying the ratio of EdU+ cells per total (DAPI+) number of cells in 10 to 20 PDOs per biological replicate. Error bars indicate mean + SD, and red line indicates median. Biological replicates: *n* = 5 (B), *n* = 2 (N), *n* = 3 [(C), (G), (I), and (L)], and *n* = 4 [(C), (F), (H), (K), and (M)]. Student’s unpaired *t* test [(B) and (K) to (N)], one-way ANOVA with Dunnett’s test [(C) and (H)], or Kruskal-Wallis with Dunn’s test [(F), (G), and (I)] was used to assess significance. Scale bars: 100 μm (D) and 40 μm [(E) and (J)]. ns, not significant.

### Catalytic activity of FAH is likely not required for FAH-mediated resistance

Given that the only known function of FAH is the conversion of fumarylacetoacetate to acetoacetate and fumarate as the last step in the tyrosine degradation pathway (fig. S1A), we evaluated whether tyrosine catabolism is enhanced in *FAH*-overexpressing cells. We therefore performed a tyrosine flux analysis, using ^13^C9-^15^N–labeled tyrosine to analyze how the tyrosine is taken up and used by the control versus FAH-high cells. Although we observed enrichment of noncanonical tyrosine catabolism metabolites such as L-DOPA and norepinephrine (fig. S3, E and F), we unexpectedly did not observe carbon labeling of the canonical tyrosine degradation pathway at 4 hours, 24 hours, or 7 days of labeling (Fig. 3, A to D; fig. S3, A to F; and data S2). This led us to speculate that the known FAH function in tyrosine catabolism or its catalytic activity is not relevant for its function as a driver of CDK4/6 resistance. To assess whether the catalytic activity is needed for the FAH-mediated resistance, we introduced two separate point mutations in the catalytic pocket: We changed H133 to alanine, which is predicted to be the site of catalytic activity (*25*), and A134 to aspartic acid, a mutation that is observed in hereditary tyrosinemia patients and causes reduced catalytic activity (*26*, *27*). Similar to wild-type (WT) FAH, the FAH H133A and A134D mutant cells showed increased resistance to abemaciclib (Fig. 3, E and F, and fig. S3, G to J), suggesting that the mutations do not perturb the noncanonical function of FAH, which is responsible for the drug resistance. Given that our data showed that the canonical role of FAH was not necessary for FAH-induced CDK4/6 resistance, we wanted to gain additional insight into FAH alternative functions. We therefore performed RNA sequencing (RNA-seq) on abemaciclib-treated empty vector (EV) and *FAH*-overexpressing MCF7 cells and observed increased transcription of genes involved in cell proliferation such as *KIFC1*, *AURKA*, and *AURKB* (Fig. 3G and data S3). Conversely, we observed decreased transcription of genes involved in cell proliferation in *FAH* knockout T47D cells (Fig. 3H and data S3). Gene set enrichment analysis revealed that cell cycle signatures were differentially enriched in the *FAH*-overexpressing cells and *FAH* knockout cells (Fig. 3, I and J, and data S3), likely reflecting the differential proliferative states of the *FAH*-overexpressing cells and knockout cells following CDK4/6 inhibitor treatment. Collectively, our data indicate that FAH drives resistance in a tyrosine catabolism–independent manner and suggest a previously unknown moonlighting function for FAH in HR+ breast cancer.

**Fig. 3.**
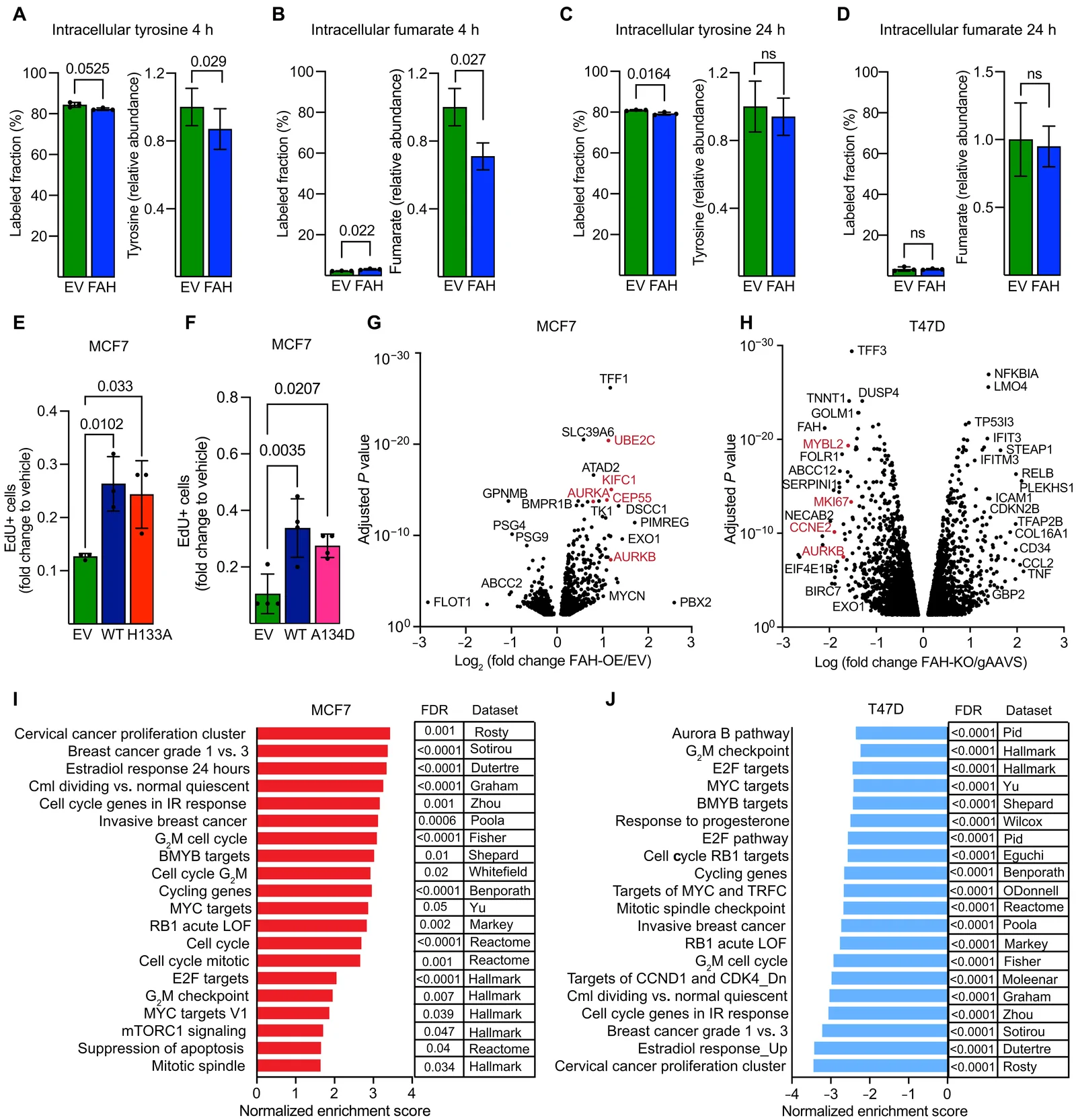
Active tyrosine degradation pathway is not necessary for FAH-mediated resistance to CDK4/6 inhibitors. (**A** and **B**) Percentage of ^13^C labeling and abundance of intracellular tyrosine (A) and intracellular fumarate (B) after 4 hours of labeling measured by liquid chromatography–mass spectrometry (LC-MS). (**C** and **D**) Percentage of ^13^C labeling and abundance of intracellular tyrosine (C) and intracellular fumarate (D) after 24 hours of labeling. Tyrosine labeling was performed by growing MCF7 EV control and *FAH*-overexpressing cells in media supplemented with 5% dialyzed fetal bovine serum (FBS) and ^13^C9 ^15^N tyrosine (0.12 g/liter) for 4 hours [(A) and (B)] or 24 hours [(C) and (D)]. (**E** and **F**) Quantification of EdU+ cells in MCF7 EV, FAH WT, and FAH H133A mutant cells (E) and FAH A134D mutant cells (F) treated with 200 nM abemaciclib for 72 hours. (**G** and **H**) Volcano plot showing enriched and suppressed transcripts in MCF7 *FAH*-overexpressing cells treated with 200 nM abemaciclib for 72 hours (G) and T47D *FAH* KO (gFAH #3) cells treated with 500 nM abemaciclib for 72 hours (H). Proliferation-related transcripts are highlighted in red. Gene set enrichment analysis of MCF7 *FAH*-overexpressing cells (**I**) and T47D *FAH* KO cells treated with abemaciclib (**J**). The data are presented as normalized enrichment scores and FDR. Error bars indicate mean + SD. Biological replicates: *n* = 3 (E) and *n* = 4 (F). Student’s unpaired *t* test [(A) to (D)] or one-way ANOVA with Dunnett’s test [(E) and (F)] was used to assess significance. h, hours.

### Nuclear localization of FAH is required for the resistance phenotype

Some metabolic enzymes can change subcellular localization and perform nonmetabolic functions such as RNA and DNA binding and affect histone and DNA methylation in the nucleus (*28*–*31*). These activities can induce transcriptional and signaling changes that allow cells to activate multigene adaptation programs and survive in changing and challenging microenvironments. Since we observed nuclear FAH staining in the PDOs that were derived from patients that relapsed after CDK4/6 inhibitor treatment (Fig. 2D), we next evaluated whether CDK4/6 inhibitors could induce FAH nuclear localization. We treated *FAH*-overexpressing MCF7 cells with abemaciclib for 72 hours and observed increased a nuclear–to–cytosolic FAH ratio (Fig. 4, A and B). Similarly, we observed increased expression of endogenous nuclear FAH in patient #10 PDOs and abemaciclib-treated T47D cells (fig. S4, A and B). This observation led us to hypothesize that nuclear FAH might play a role in the development of therapy resistance. To test this hypothesis, we added a nuclear localization signal (NLS) or a nuclear export signal (NES) to our *FAH* construct and overexpressed these in MCF7 cells and patient #26 PDOs (Fig. 4C and fig. S4C). Similar to WT FAH, the FAH-NLS cells had increased proliferation (EdU+ cells), whereas the FAH-NES cells were equally sensitive to abemaciclib as the EV control cells (Fig. 4, D to F, and fig. S4, E to H). To evaluate the presence of nuclear FAH in breast cancer patient tumors, we scored both cytoplasmic and nuclear expression of FAH in 12 matched pre- and post-CDK4/6 inhibitor–treated patient tumors (data S1). While the overall expression of FAH did not increase in the posttreatment tumors, nuclear FAH did: ∼58% of the tumors in the pretreatment group were positive for nuclear FAH, and this notably increased to ∼91% in the posttreatment group (Fig. 4, G and H). These data show that nuclear localization is essential for FAH-mediated resistance to CDK4/6 inhibitors and that nuclear FAH could play a role in CDK4/6 inhibitor resistance and may represent a biomarker for treatment resistance.

**Fig. 4.**
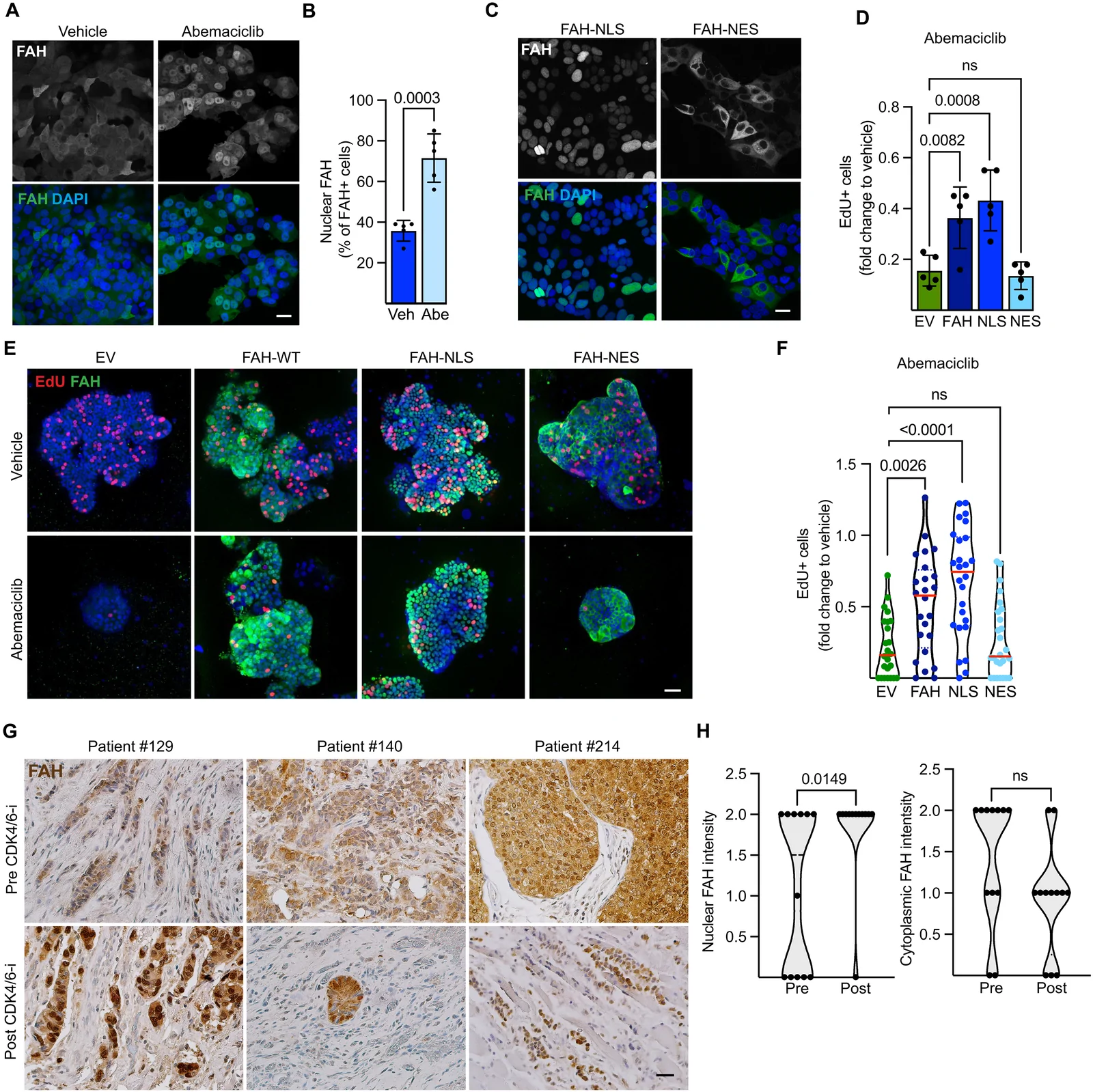
Nuclear localization of FAH is required for FAH-mediated resistance to CDK4/6 inhibitors. (**A** and **B**) Representative confocal images of FAH immunofluorescence staining (A) and quantification (B) of nuclear FAH in MCF7 *FAH*-overexpressing cells treated with 200 nM abemaciclib for 72 hours. (**C** and **D**) Representative confocal images (C) of FAH immunofluorescence in MCF7 NLS (FAH-NLS) and FAH nuclear export signal (FAH-NES) cells and (D) quantification of EdU+ cells in MCF7 EV control, FAH-WT, FAH-NLS, and FAH-NES cells treated with 200 nM abemaciclib for 72 hours. Proliferation was assessed by pulsing cells for 2 hours with EdU. (**E** and **F**) Representative confocal images EdU and FAH staining (E) and quantification (F) of EdU+ cells in patient #26 EV, FAH-WT, FAH-NLS, and FAH-NES expressing PDOs treated with 300 nM abemaciclib for 7 days. Proliferation was assessed by pulsing PDOs with EdU for 4 hours and quantifying the ratio of EdU+ cells per total (DAPI+) number of cells in 15 to 20 PDOs from each biological replicate. (**G** and **H**) Representative images of FAH immunohistochemistry (G) and scoring of nuclear and cytoplasmic FAH (H) of patient tumors pre- and post-CDK4/6 inhibitor treatment. Student’s unpaired *t* test (B), one-way ANOVA with Dunnett’s test [(D) and (F)], or Wilcoxon rank sum test, using the Holm-Bonferroni correction method (H), was used to assess significance. Error bars indicate mean + SD, and red line indicates median. Biological replicates: *n* = 5 [(B) and (D)] and *n* = 2 (F). Scale bars, 20 μm [(A) and (C)] and 40 μm [(E) and (G)].

### FAH interacts with CDK9

To explore the nuclear functions of FAH, we used a proximity labeling assay to identify nuclear binding partners of FAH. To do this, we cloned our FAH-NLS and FAH-NES constructs into the BioID vector that enables labeling and isolation of biotinylated proteins that are close by or in direct interaction with FAH (*32*). We first confirmed by streptavidin staining that the biotin-treated MCF7 FAH-NLS BioID and FAH-NES BioID cells express biotinylated proteins in the correct cellular locations and that the FAH-NLS BioID cells are more resistant to abemaciclib (fig. S5, A and B). Next, we treated the cells with abemaciclib for 72 hours and added biotin for the last 24 hours and performed streptavidin pulldown of biotinylated proteins followed by mass spectrometric analysis. We observed that FAH interacts with several proteins involved in DNA, histone, and chromatin binding (Fig. 5, A and B; fig. S5C; and data S4). We found FAH interactions with transcriptional regulatory CDKs such as CDK9 that has shown promise as a therapeutic target in CDK4/6 inhibitor–resistant HR+ breast cancer in preclinical studies (*33*). To validate the FAH-CDK9 interaction, we performed coimmunoprecipitations and proximity ligation assays (PLAs) that allowed us to visualize and quantify subcellular localization of protein-protein interactions in situ. Since the *FAH* overexpression construct is tagged with V5, we used V5-tag for our protein-protein interaction assays. We observed that FAH-V5 and CDK9 interact in both vehicle- and CDK4/6 inhibitor–treated conditions and that the interaction increases during abemaciclib treatment (Fig. 5,C and D, and fig. S5D). To evaluate whether FAH increases the transcriptional activity of CDK9, we assessed induction of the CDK9 target genes C-MYC and B-MYB in PDOs overexpressing *FAH*. We observed a significant increase in *C-MYC* mRNA expression in *FAH*-overexpressing PDOs in both vehicle- and abemaciclib-treated PDOs and *B-MYB* in abemaciclib-treated PDOs (fig. S5E). Similarly, C-MYC and B-MYB targets were among the top positively enriched gene sets in abemaciclib-treated *FAH*-overexpressing MCF7 cells and negatively enriched targets in T47D *FAH* knockout cells (Figs. 3, I and J, and 5, E and F). Furthermore, we confirmed increased C-MYC protein levels in *FAH*-overexpressing MCF7 cells and PDOs and decreased C-MYC protein in T47D *FAH* knockout cells (Fig. 5, G to I), suggesting that FAH increases the CDK9 pathway activity.

**Fig. 5.**
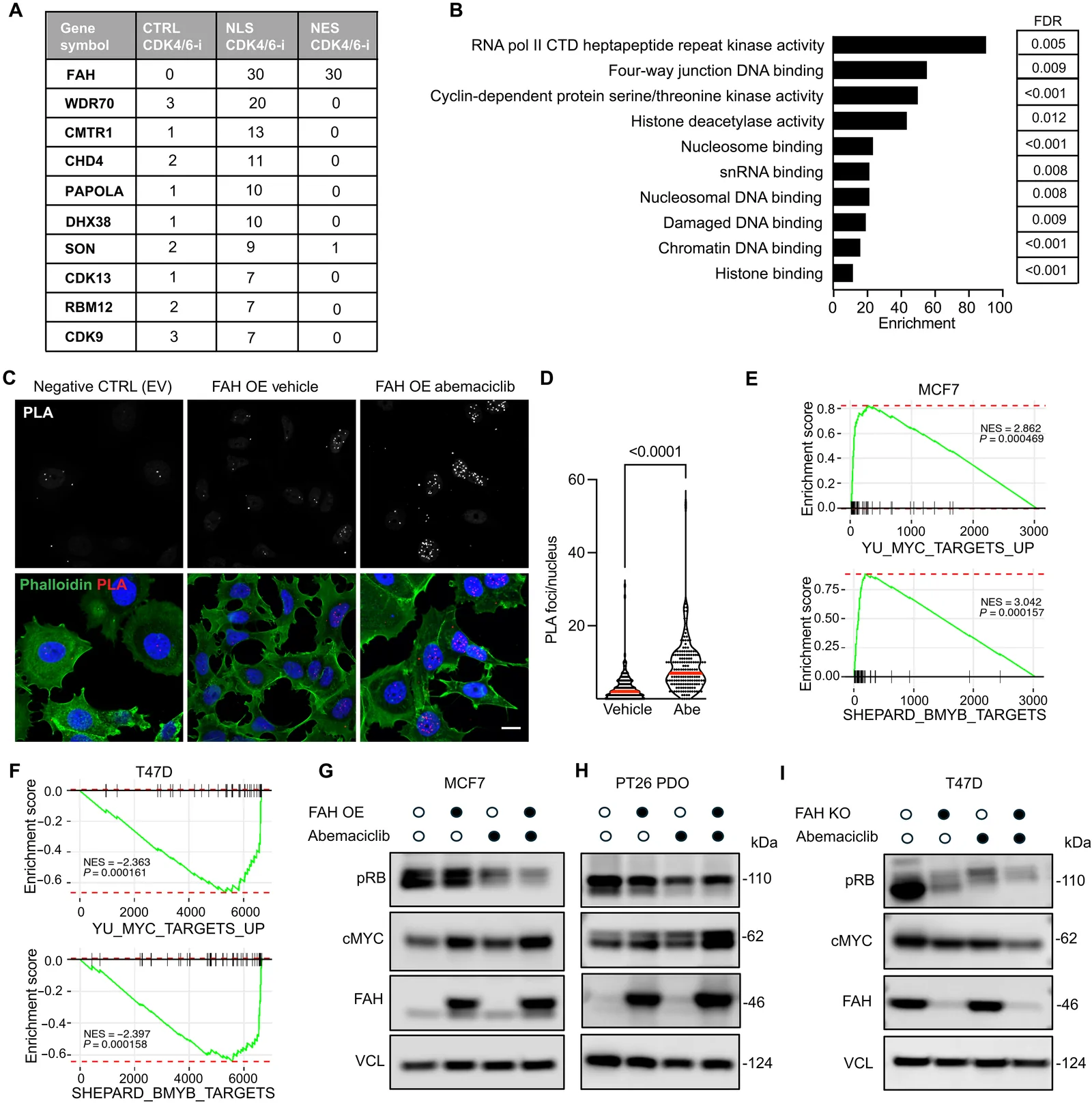
FAH directly interacts with CDK9. (**A**) Table with top 10 potential FAH interactors in MCF7 FAH-NLS BioID cells treated with 200 nM abemaciclib. Table shows average peptide counts from triplicate samples analyzed by mass spectrometry. CTRL BioID and FAH-NES BioID peptide counts are shown as controls. (**B**) Bar chart of GO molecular function pathway enrichment of proteins interacting with FAH-NLS under abemaciclib treatment. Data are plotted as fold enrichment of GO terms that are overrepresented in the dataset compared to the background set and FDR of the enrichment. snRNA, small nuclear RNA. (**C** and **D**) Representative images (C) and quantification (D) of nuclear PLA foci in vehicle- and abemaciclib-treated MCF7 FAH-V5 cells. PLA was used to assess FAH (V5-tag) and CDK9 interaction in 100 cells. (**E** and **F**) Enrichment score of MYC targets and B-MYB targets in MCF7 EV and FAH-OE cells (E) and T47D gAAVS and gFAH#3 KO cells (F) treated with abemaciclib for 72 hours. Yu MYC targets up and Shepard BMYB targets through the mSigDB were used for the analysis. (**G** to **I**) Western blot analysis of pRB, cMYC, FAH, and vinculin (VCL) protein in vehicle- and abemaciclib-treated MCF7 EV control and *FAH*-expressing cells (G), patient #26 EV and FAH PDOs (H) and T47D gAAVS and gFAH #3 KO cells (I). Biological replicates: *n* = 2 (D). Red line indicates median. Mann-Whitney test was used to assess significance. Scale bar, 10 μm.

### FAH-mediated resistance is conveyed via CDK9

To assess whether the interaction with CDK9 is essential for FAH-mediated resistance, we sought to disrupt the interaction. We first used AlphaFold to perform computational prediction of interaction-essential sites and identified CDK9 Q25 and FAH D143 as a putative site of interaction (Fig. 6A). We next mutated the D143 site by changing the aspartic acid to lysine and confirmed that FAH D143K is stably expressed and displays nuclear localization following abemaciclib treatment (Fig. 6B and fig. S6A). Next, we evaluated the FAH and CDK9 interaction by PLA on vehicle- and abemaciclib-treated FAH-WT and FAH D143K expressing MCF7 cells. Since the catalytic site mutants do not interfere with FAH-mediated resistance, we included FAH H133A and FAH A134D as positive controls. We observed a significant increase in nuclear CDK9-FAH PLA foci in abemaciclib-treated FAH WT, FAH H133A, and A134D cells, and this was significantly decreased in the FAH D143K expressing cells (Fig. 6, C and D, and fig. S6, B and C), showing that mutating the D143 site significantly disrupts the FAH-CDK9 interaction. Since the D143K cells have decreased ability to interact with CDK9, we next treated EV, FAH WT, and FAH D143K cells with increasing doses of abemaciclib. Notably, we observed that the FAH D143K cells were equally sensitive to abemaciclib as the EV cells (Fig. 6E and fig. S6D), indicating that the reduced interaction with CDK9 is enough to reverse the FAH-mediated resistance. Next, we evaluated whether FAH D143K cells have decreased expression of CDK9 target gene C-MYC. We observed decreased expression of C-MYC protein in FAH D143K cells (Fig. 6F), suggesting that the CDK9 pathway activity is decreased in the FAH D143K mutant cells. Last, to further evaluate whether this interaction drives FAH-mediated resistance, we treated FAH-overexpressing PDOs with a CDK9 inhibitor AZD4573. This CDK9 inhibitor is being evaluated in clinical trials for hematologic malignancies and was shown effective when combined with palbociclib and fulvestrant (ER degrader) in preclinical experiments in ER-positive breast cancer (*33*). We used AZD4573 alone or in combination with abemaciclib. The *FAH*-overexpressing PDOs showed increased sensitivity to AZD4573 alone, and the combination treatment with CDK9 and CDK4/6 inhibitors induced robust apoptosis (Fig. 6, G to I, and fig. S6E). These data collectively suggest that the FAH-mediated resistance is conveyed via its interaction with CDK9.

**Fig. 6.**
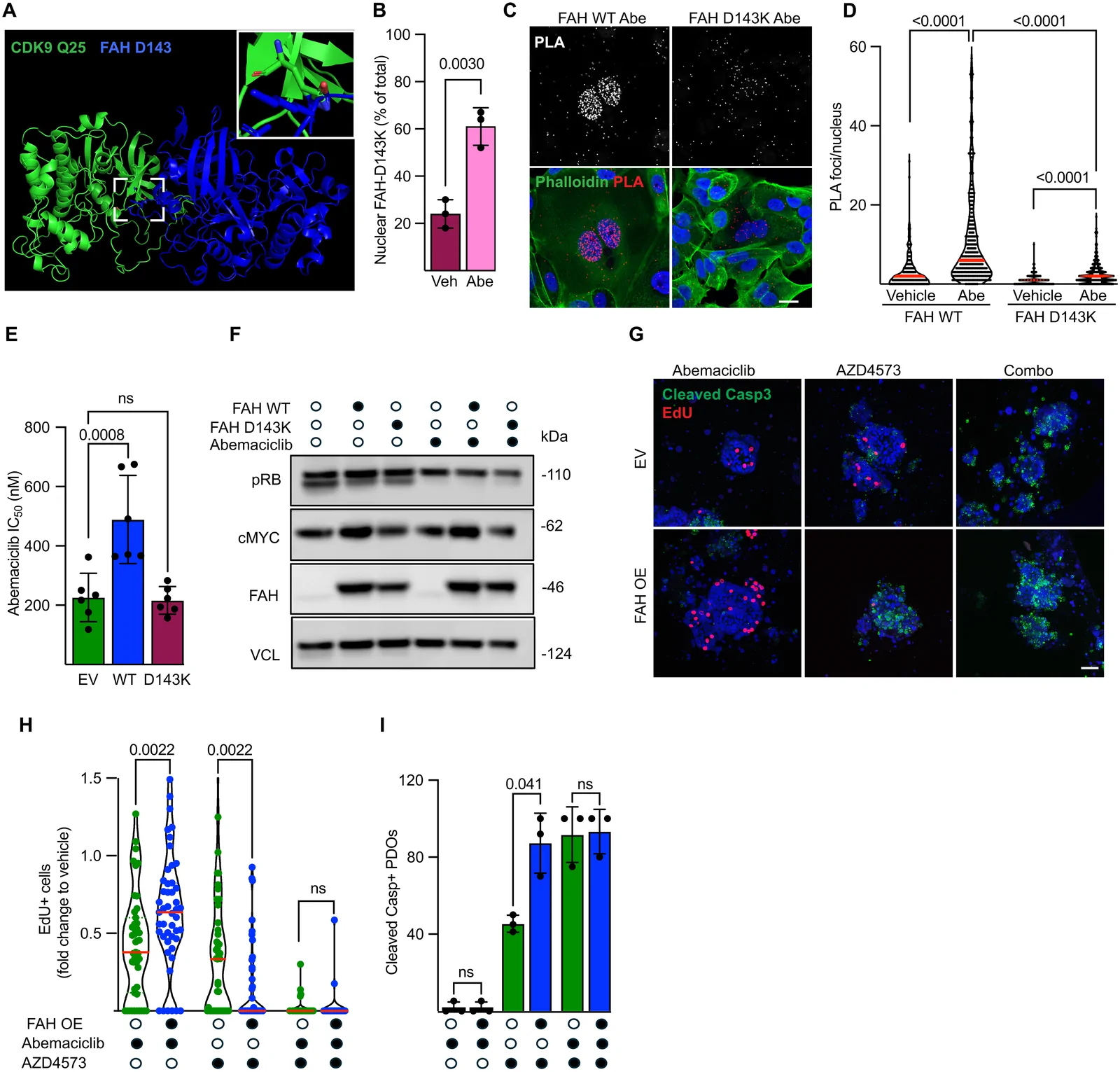
FAH-mediated resistance is conveyed via CDK9. (**A**) AlphaFold prediction of FAH-CDK9 interaction. AlphaFold multimer prediction was performed using the Cosmic^2^ platform. (**B**) Quantification of nuclear FAH-D143K in MCF7 cells treated with vehicle or 200 nm abemaciclib for 72 hours. (**C** and **D**) Representative images (C) and quantification (D) of PLA foci in vehicle- and abemaciclib-treated MCF7 FAH-WT-V5 and FAH D143K-V5 cells. PLA was used to assess V5-tag and CDK9 interaction in 100 cells. (**E**) Abemaciclib IC_50_ of MCF7 EV, FAH-WT, and FAH D143K cells treated with increasing doses of abemaciclib for 72 hours. PrestoBlue cell viability reagent was used to assess cell viability. (**F**) Western blot analysis of pRB, cMYC, FAH, and vinculin (VCL) protein in vehicle- and abemaciclib-treated MCF7 EV control, FAH-WT, and FAH-D143K cells. (**G**) Representative confocal images of cleaved caspase 3 and EdU staining, (**H**) quantification of EdU+ cells, and (**I**) cleaved caspase 3+ PDOs in patient #26 PDOs treated with vehicle, 300 nM abemaciclib, 15 nM AZD4573 (CDK9 inhibitor), or the combination of abemaciclib and AZD4573 for 96 hours. Proliferation was assessed by pulsing PDOs with EdU for 4 hours and quantifying the ratio of EdU+ cells per total (DAPI+) number of cells. Apoptosis was quantified by staining for cleaved caspase 3–positive PDOs. Ten to 15 PDOs were analyzed per each individual experiment. Biological replicates: *n* = 3 (D), *n* = 5 (E), and *n* = 3 [(H) and (I)]. Error bars indicate mean + SD, and red line indicates median. Student’s unpaired *t* test [(H) and (I)], one-way ANOVA with Dunnetts’ test (E), or Kruskal-Wallis with Dunn’s test (D) was used to assess significance. Scale bars, 10 μm (C) and 40 μm (H).

## DISCUSSION

Here, we suggest a previously unknown function for the enzyme FAH in HR+ breast cancer. We show that *FAH* is up-regulated in HR+ breast cancer where it induces resistance to CDK4/6 inhibitors and correlates with poor survival. By using archived patient tumors and HR+ PDOs, we show that FAH translocates to the nucleus upon CDK4/6 inhibitor treatment. Blocking the nuclear translocation reverses the FAH-mediated resistance, suggesting that nuclear FAH is essential for CDK4/6 inhibitor resistance. We further show that the FAH’s function as a metabolic enzyme in the tyrosine degradation pathway is not manifested in the HR+ breast cancer cells as the canonical tyrosine catabolism pathway is not active in these cells based on our tracing data, and therefore FAH’s catalytic activity is most likely not required for the resistance phenotype. Our experiments with the 4-hydroxyphenylpyruvate dioxygenase (HPD) inhibitor nitisinone further support this conclusion, as cells treated with the tyrosine catabolism inhibitor still showed resistance toward CDK4/6 inhibition. Mechanistically, we showed that FAH interacts with CDK9 and increases CDK9 target genes and that disruption of the FAH-CDK9 interaction reverses FAH-mediated resistance. Furthermore, by treating PDOs with a CDK9 inhibitor, we show that *FAH*-overexpressing PDOs exhibit increased sensitivity to CDK9 inhibition, whereas the combination treatment induces large-scale apoptosis of HR+ PDOs, reversing the FAH-mediated resistance.

FAH is the last enzyme in the tyrosine degradation pathway where it breaks down fumarylacetoacetate to fumarate and acetoacetate. The function of FAH was mostly described in studies that focus on hereditary tyrosinemia 1 (HT1), where its decreased enzymatic function causes accumulation of toxic upstream metabolites leading to progressive renal and liver damage, neurological crises, and hepatocellular carcinoma if left untreated (*27*, *34*). Apart from its function in HT1 and hepatocellular carcinoma, FAH is up-regulated in melanoma where it promotes tumor growth and migration by inducing metabolic reprogramming and in ovarian cancer where its decreased function drives chemoresistance (*35*, *36*). Although these studies describe multiple functions for FAH, tyrosine catabolism and/or the catalytic activity of FAH is required for the described functions. Unexpectedly, we could not detect labeling of the canonical tyrosine degradation pathway following a tracing experiment that was carried on for 4 hours and up to as long as 7 days of exposure to stable isotope–labeled tyrosine. This and the fact that perturbation of the tyrosine degradation pathway using the predicted catalytic site mutant H133A as well as the patient mutation A134D did not affect the FAH-induced resistance collectively show that tyrosine degradation is most likely not involved in FAH’s role in CDK4/6 inhibitor resistance. However, the tyrosine metabolism pathway is enriched in breast cancer, and we observed increased levels of metabolites of the side branches, thereby the pathway may contribute to other aspects of HR+ breast cancer biology.

In recent years, noncanonical functions of metabolic enzymes have gained substantial research focus as several enzymes have been described to perform previously unknown functions beyond the classical metabolic pathways. These “moonlighting” functions are not limited to metabolic functions but can also perform unexpected activities such as regulation of transcription (*31*). In addition, some metabolic enzymes change their spatial location in response to different cellular stressors. Starvation can induce nuclear localization of acetyl-CoA synthetase 2 where it interacts with transcription factor EB and activates transcription of lysosomal and autophagosome genes, ultimately leading to cell growth and survival of glioblastoma cells (*37*). DNA damage can induce nuclear localization of fumarate hydrolase where increased levels of fumarate promote DNA repair via chromatin remodeling (*38*, *39*). Li *et al.* (*36*) demonstrated that under genotoxic chemotherapy, FAH gets oxidized and translocates to the nucleus where it performs tyrosine degradation and sensitizes ovarian cancer cells to chemotherapy. This study supports our finding that FAH can translocate to the nucleus in response to CDK4/6 inhibition. However, unlike Li *et al.* (*36*), FAH-expressing breast cancer cells do not use tyrosine degradation. Furthermore, it is unlikely that the mechanism for nuclear localization is similar in our system, as our PDO models have strong nuclear expression of FAH despite the media being supplemented with the antioxidant *N*-acetyl-l-cysteine that reverses the nuclear translocation in the ovarian cancer cell lines used by Li *et al.* (*36*).

In our hands, FAH interacted with CDK9, and FAH-expressing cells showed increased sensitivity to CDK9 inhibition. CDK9 is a member of the transcription-associated CDKs, and it is a key component of the positive transcription elongation factor b complex that releases paused RNA polymerase II to promote elongation. The CDK9 pathway is implicated in tumorigenesis of multiple hematological malignancies and solid tumors, including breast cancer (*40*). Increasing evidence suggests that CDK9 activity is required for the maintenance of sufficient levels of short-lived transcripts such as the proto-oncogenes *C-MYC* and *MYB* that are known to drive tumor growth and resistance to multiple targeted therapies, including CDK4/6 inhibitors (*41**–**45*). In HR+ breast cancer, CDK9 inhibition suppresses MYC and MYB in cell lines and in patient-derived xenografts (PDXs) (*33*, *46*, *47*). We also observed increased expression of *C-MYC* and *MYB* in our *FAH*-expressing models, particularly during CDK4/6 inhibition, further supporting our data showing that FAH activates the CDK9 pathway to drive resistance. It is reported that PDX models derived from ET and palbociclib-resistant metastasis show increased sensitivity to CDK9 inhibition in combination with targeted therapies (*33*), similar to our findings in *FAH*-expressing PDOs. Because our work evaluated this combination treatment in *FAH*-overexpressing PDOs, future studies using single-agent CDK9 inhibition or combination treatment in additional FAH-high PDOs and in vivo are needed to evaluate the efficacy and safety of this combination. However, the incentive for these studies is high because our preliminary PDO data suggest that patients that have relapsed on ET + CDK4/6-i and express nuclear FAH could benefit from single-agent CDK9 inhibition or combination therapy with CDK4/6 inhibitors. Further, these findings might be of relevance to additional breast cancer subtypes, such as HER2 positive: In addition to the high FAH levels in these tumors, high CDK9 mRNA correlates with poor survival, and MYC was reported to drive resistance toward HER2 targeted therapies (*48*, *49*). This implies that the pathway of FAH-CDK9 and downstream MYC is active in HER2-positive tumors and that the CDK9-MYC arm is driving tumor progression or drug resistance. Therefore, it is plausible that the FAH-CDK9 pathway has a broader function in driving resistance, and targeting this pathway could be beneficial also to HER2-positive breast cancers.

Collectively, our work reveals a previously unknown noncatalytic function for FAH in breast cancer, offering two clinically relevant routes: the possibility of the utilization of nuclear FAH as a biomarker of resistance to CDK4/6 inhibitors and the molecular insight of this unexpected resistance mechanism. Notably, this insight suggests CDK9 inhibition alone or in combination with CDK4/6 inhibitors as an immediate, actionable clinical approach for HR+ breast cancer patients expressing nuclear FAH. Therefore, the impact of our work is tangible and substantial, and future work will be of interest for clinicians and can benefit CDK4/6-inhibitor treated HR+ breast cancer patients. 

## MATERIALS AND METHODS

### Cell culture of breast cancer cell lines

MCF7, MDA-MB-175, and T47D cells were a gift from J. Brugge (Harvard Medical School, Boston, MA) and were authenticated by STR analysis before experiments. Human embryonic kidney (HEK) 293T cells were from American Type Culture Collection. Cells were mycoplasma tested once a month (MycoAlert Mycoplasma detection kit, Lonza) and grown in Dulbecco’s modified Eagle’s medium (DMEM) media with 10% fetal bovine serum (FBS) and penicillin/streptomycin (+37°C, 5% CO_2_).

### Plasmids, cloning, CRISPR-Cas9 KO, site-directed mutagenesis

LentiCRISPR v2 was a gift from F. Zhang (Addgene plasmid #52961) (*50*). The constructs for *FAH* KO were created by annealing the following sense and antisense oligonucleotides: KO-1s CACCGTGTAGGCTAAGACCAAAAC, KO-1as AAACGTTTTGGTCTTAGCCTACAC, KO-2s CACCGTCTGGTCGAGAAGACGCCGT, KO-2as AAACACGGCGTCTTCTCGACCAGAC, KO-3s CACCGCGGCAGCATGCTACCAACGT, and KO-3as AAACACGTTGGTAGCATGCTGCCGC, followed by subcloning in the LentiCRISPR v2 backbone. Constructs to create control cells were created by annealing the following sense and antisense oligonucleotides: one missense sequence targeting bacterial *LacZ*-s CACCTGCGAATACGCCCACGCGAT and *LacZ*-as AAACATCGCGTGGGCGTATTCGCA. Coding sequence of *FAH* was ordered from GenScript and further subcloned into pLenti6/V5-*p53_wt p53* (gift from B. Futscher, Addgene plasmid #22945) (*51*) by cutting out the coding sequence of *p53* and pasting the coding sequences of FAH into via *Xba*I and *Spe*I restriction sites, allowing the generation of the C-terminal V5-tagged *FAH* construct.

To generate mutations in the catalytic residues of *FAH* or the FAH-CDK9 interaction site, site-directed mutagenesis reactions were performed following the QuikChange II XL Site-Directed Mutagenesis Kit protocol (Agilent, #200521). The following primers were generated using Agilent’s QuikChange Primer Design tool: H133A, 5′ TTCTATTCCTCTCGGCAGGCTGCTACCAACGTCGGAATC (forward) and 5′ GATTCCGACGTTGGTAGCAGCCTGCCGAGAGGAATAGAA (reverse); D143K, 5′CGTCGGAATCATGTTCAGGAAGAAGGAGAATGCGTTGATGCCAAA (forward) and 5′TTTGGCATCAACGCATTCTCCTTCTTCCTGAACATGATTCCGACG (reverse); A134D, 5′ CGGCAGCATGATACCAACGTCGGAATCATGTTC (forward) and 5′ GAACATGATTCCGACGTTGGTATCATGCTGCCG (reverse).

To generate *FAH*-NLS and *FAH*-NES constructs, SpeI and ApaI flanked *FAH* fragments containing C-terminal NLS or NES sequences were generated by amplifying the *FAH* sequence from pLenti6-V5-*FAH* using two sequential rounds of polymerase chain reaction (PCR) with the *FAH* NLS/NES primers (*FAH*-NLS-F′-round1: GATCCACTAGTGCCTCCATGTCCTTC, *FAH*-NLS-R′-round1: CACGCGTTTCGCCGCCGGGGCTGATGGCAGGAGAGCAG, *FAH*-NLS-F′-round2: same primer as *FAH*-NLS-F′-round1, *FAH*-NLS-R′-round2: ATCGGGCCCGAATCCAGTTTCACGCGTTTCGCCGCC, *FAH*-NES-F′-round1: same primer as *FAH*-NLS-F′-round1, *FAH*-NES-R′-round1: GCGCTCCAGGGGGGGCAGGGCTGATGGCAGGAGAGCAG, *FAH*-NES-F′-round2: same primer as *FAH*-NLS-F′-round1, *FAH*-NES-R′-round2: ATCGGGCCCGACAGGGTCAGGCGCTCCAGGGGGG). *FAH*-NLS and *FAH*-NES PCR fragments and pLenti6-V5-*FAH* were digested using SpeI and ApaI and ligated to generate pLenti6-V5-*FAH*-NLS and pLenti6-V5-*FAH*-NES plasmids.

To generate the *FAH* BioID constructs, *Bam*HI and *Eco*RI flanked *FAH* fragments were PCR amplified and subcloned into mycBioID2-pBABE-puro (gift from K. Roux, Addgene #80900, no longer available on Addgene). For the *FAH*-WT-BioID construct, PCR amplification was performed using pLenti6-V5-*FAH* as the template with *FAH*-WT-BioID primers (*FAH*-WT-BioID-*Bam*HI-F′: GTATACTGGATCCGCCGCCACCATGTCCTTCATCCCGG, *FAH*-WT-BioID-*Eco*RI-R′: GGCGGTGAATTCTGATGGCAGGAGAGCA). For the *FAH*-NLS-BioID construct, PCR amplification was performed using pLenti6-V5-*FAH*-NLS as the template with *FAH*-NLS-BioID primers (*FAH*-NLS-BioID-*Bam*HI-F′: same as *FAH*-WT-BioID-*Bam*HI-F′, *FAH*-NLS-BioID-*Eco*RI-R′: GGCGGTGAATTCATCCAGTTTCACGCGT). For the *FAH*-NES-BioID construct, PCR amplification was performed using pLenti-V5-*FAH*-NES as the template with *FAH*-NES-BioID primers (*FAH*-NES-BioID-*Bam*HI-F′: same as *FAH*-WT-BioID-*Bam*HI-F′, *FAH*-NES-BioID-*Eco*RI-R′: GGCGGTGAATTCCAGGGTCAGGCGC). *FAH*-WT-BioID, *FAH*-NLS-BioID, and *FAH*-NES-BioID PCR fragments and mycBioID2-pBABE-puro were digested using *Bam*HI and *Eco*RI and ligated to generate the mycBioID2-pBABE-*FAH*-WT, mycBioID2-pBABE-*FAH*-NLS, and mycBioID2-pBABE-*FAH*-NES plasmids.

### Transfection and lentiviral transduction

HEK293T cells were seeded on 6-cm plates and transfected with Lipofectamine 3000 reagents the next day as follows. A master mix of 3 μg of target plasmid DNA, 2.2 μg of psPAX2 (gift from A. Toker, BIDMC), 0.8 μg of VSVG (gift from J. Brugge, HMS), 10 μl of P3000 reagent, and 700 μl of OptiMEM (Thermo Fisher Scientific, #31985070) was mixed with 700 μl of OptiMEM and 20 μl of Lipofectamine 3000 reagent (Thermo Fisher Scientific # L3000015) and incubated for 20 min at room temperature. The mixture was added dropwise to HEK293T cells and replaced the next day with fresh DMEM containing 10% FBS. Lentivirus was harvested 48 and 72 hours after transfection, filtered through a 0.45-μm filtered, and concentrated using the PEG-it virus precipitation protocol (System Biosciences, #LV810A-1). Breast cancer cells were seeded on 6-cm plates and incubated overnight with concentrated lentivirus and polybrene (5 μg/ml; Sigma-Aldrich, 107689). Transduced breast cancer cells were selected with either puromycin (10 μg/ml; Thermo Fisher Scientific, #A1113803) for 3 days or blasticidin (10 μg/ml; Thermo Fisher Scientific, #A1113903) for 7 days. To transduce PDO, PDO fragments were resuspended in PDO media supplemented with polybrene (5 μg/ml), 10 μM Y-27632 (SelleckChem, #S1049), and concentrated lentivirus and incubated overnight on a low adhesion plated. The following day, the PDOs were pelleted and embedded in 50 μl of Cultrex on a 24-well plate. To select for transduced cells, puromycin (5 μg/ml) was added for 3 days or blasticidin (10 μg/ml) for 7 days.

### Proliferation and cell viability assays

To assess the IC_50_ value of abemaciclib, breast cancer cells were seeded at 12,000 cells per well at a 96-well plate. The following day, the media was replaced with DMEM containing 1% FBS, and cells were treated with increasing concentrations of abemaciclib (SelleckChem, #S5716) ranging from 0.05 to 10 μM. Cell viability was analyzed at 72 hours with PrestoBlue HS cell viability reagent (Thermo Fisher Scientific, #P50201). To assess cell proliferation, MCF7 cells were seeded on fibronectin-coated coverslips at 100,000 cells per well on a 24-well plate (Thermo Fisher Scientific, #142475). The following day, the media was replaced with DMEM containing 1% FBS, and cells were treated with 200 nM abemaciclib. After 72 hours of treatment, cells were pulsed with 10 μM EdU for 2 hours and fixed with 4% paraformaldehyde (PFA) for 10 min at room temperature.

### Patient material used in the study

Tissues for evaluating FAH expression pre- and post-CDK4/6 inhibition were from 12 patients diagnosed with HR+ breast cancer between 1999 and 2021. The patient tumor collection includes treatment-naïve/ET only tumors and matching metastasis treated with ET and palbociclib. The specimens were collected according to protocol DF/HCC (#) 17-503 approved by the Institutional Review Board (IRB) at Dana Farber Harvard Cancer Center. Specimens were deidentified, logged into the RedCap database, and retrieved from the pathology archive. The DF/HCC IRB Protocol (#) 17-627 and procedure to collect biopsies for PDO cultures was described by Hogstrom *et al.* (*52*). Informed consent was obtained from all participants per Federal Regulations (45 CFR 46), BIDMC IRB Guidelines and requirements of HIPAA, and the studies abide by the Declaration of Helsinki principles.

### PDO cultures

Propagation, culturing, and drug sensitivity analysis of PDO cultures were previously described (*52*, *53*). Briefly, PDOs were incubated in 1× Dispase-II solution (Sigma-Aldrich, #SCM133) with collagenase (2 mg/ml) for 30 to 45 min at 37°C and mechanically disrupted by passing through a 26G needle. PDOs were washed once with Advanced DMEM/F12 (Thermo Fisher Scientific, #12634010) supplemented with 5% FBS and pelleted by centrifugation at 400*g* for 5 min. PDO fragments were embedded in Cultrex growth factor–reduced basement membrane extract type II (Biotechne, 3532-005-02), 50-μl drops were plated into a 24-well plate, and 500 μl of PDO media or bone metastasis PDO media was added 30 min later. To analyze drug sensitivity to CDK4/6 inhibitors, PDO fragments were plated into four-well or eight-well chamber slides at 200 to 600 fragments per well, and PDOs were treated with 300 nM abemaciclib and 15 nM AZD4573 (Selleckchem, #S8719) for 4 days or 300 nM abemaciclib or palbociclib (Selleckchem, #S4482) for 7 days. To analyze sensitivity to CDK9 inhibition or dual CDK4/6 and CDK9 inhibition, PDOs were treated for 4 days with 300 nM abemaciclib and 15 nM AZD4573 that has been used in preclinical studies (*33*). On the last day, PDOs were pulsed with 10 μM EdU for 4 to 6 hours and fixed with 4% PFA for 30 min.

### Immunohistochemistry

PDOs and patient tumor sections were deparaffinized and treated with low pH citrate buffer in a microwave at full power for 2 min and then 10 min at low power. Immunohistochemistry for FAH (Sigma-Aldrich, #HPA041370) was performed using the ImmPRESS Excel Amplified Polymer Staining kit (Vector Laboratories, #MP-7601) according to the manufacturer’s description. The sections were imaged using an Olympus bright-field microscope. The tissue sections were scored by a board-certified pathologist for FAH intensity and localization. The staining intensity was categorized by relative intensity by a breast cancer pathologist as follows: 0: negative; 1: weak; 2: moderate/strong. Statistical analysis was performed using the Holm-Bonferroni method, and values lower than 0.05 were considered significant. Breast cancer TMA BR1921c with 192 cores representing 189 total cases with 21 adjacent normal tissue, 4 cancer adjacent tissue, and 7 normal tissue cores, including TNM, clinical stage and HR, and HER2 marker, was purchased from US Biomax Inc. TMA sections were scored by a breast cancer pathologist for relative FAH intensity as follows: 0: negative; 1: weak; 2: moderate, 3: strong.

### Whole-mount staining

Whole-mount staining was performed according to a previously published method (*52*): Fixed PDOs were washed with phosphate-buffered saline (PBS) and permeabilized with wash buffer (0.3% Triton X-100 in PBS) for 20 min. EdU labeling was performed for 40 min using the EdU Click-IT imaging kit Alexa Fluor 594 (Invitrogen) according to the manufacturer’s instructions. PDOs were washed three times with wash buffer, blocked for 1 hour with blocking buffer [5% goat serum, 1% bovine serum albumin (BSA), and 0.3% Triton X-100 in PBS], and incubated with anti-FAH antibody (Sigma-Aldrich, #HPA041370) at 4°C overnight in blocking buffer. The following day, the PDOs were washed 10 times with wash buffer, incubated with goat-anti rabbit Alexa Fluor 488 secondary antibody (Thermo Fisher Scientific, #A-11008) and Hoechst for 2 hours at room temperature, washed extensively with wash buffer, and mounted using Vectashield mounting media containing 4′,6-diamidino-2-phenylindole (DAPI; Vector Laboratories, #H-1800-2). PDOs were imaged with a Zeiss LSM 880 confocal microscope. To assess proliferation, equal numbers of PDOs per treatment arm were imaged, and the ratio of EdU-positive cells per total number of cells was quantified. For each experiment, technical replicates were quantified as following: 10 patient #8 PDOs, 15 patient #10 PDOs, 15 to 20 patient #26 PDOs, and 10 to 15 patient #27 PDOs. Each experiment was repeated once with patient #27 PDOs and two to four times with patient #8, #10, and #26 PDOs.

### Immunofluorescence staining

Fixed cells were washed with PBS and permeabilized with 0.3% Triton X-100 in PBS for 10 min. EdU labeling was performed for 20 min using the EdU Click-IT imaging kit Alexa Fluor 594 (Invitrogen) according to the manufacturer’s instructions. Cells were blocked for 15 min with blocking buffer (5% goat serum, 1% BSA, and 0.1% Triton X-100 in PBS) and incubated overnight at 4°C with primary antibodies diluted in 1% BSA and 0.1% Tx-100 in PBS. Cells were incubated with anti-FAH (Sigma-Aldrich, #HPA041370) diluted in blocking buffer overnight at 4°C, washed with PBS, incubated with secondary antibodies (Alexa 488) for 1 hour at room temperature, and mounted using Vectashield mounting media containing DAPI. Cells were imaged with a Zeiss LSM 880 confocal microscope.

### Proximity ligation assay

The FAH-CDK9 interaction was detected with the Duolink PLA Kit (DUO92102, Sigma- Aldrich) according to the manufacturer’s protocol. MCF7 EV (negative ctrl), FAH WT, FAH H133A, FAH A134D, and FAH D143K cells were seeded on coverslips and treated with abemaciclib for 72 hours. Coverslips were fixed with 4% PFA for 10 min, permeabilized for 15 min with 0.1% Tx-100 in PBS, and blocked for 1 hour at 37°C using the Duolink blocking buffer. Coverslips were incubated overnight with rabbit CDK9 antibody (CST, #2316) and mouse V5-tag antibody (Thermo Fisher Scientific, #R960-25). On the next day, cells were incubated with Duolink probes, ligation solution, and amplification solution. After the last wash with 0.1% buffer B, coverslips were incubated with phalloidin (Invitrogen, #A12379) for 60 min at room temperature. Last, to visualize nuclei, cells were incubated with bisbenzimide (1:5000, Hoechst stain, Sigma-Aldrich) for 10 min at room temperature, and cells were mounted using Epredia Immumount (Thermo Fisher Scientific, #9990402). Coverslips were imaged with a Zeiss LSM 880 confocal microscope 63× PlanApo oil immersion objective. Nuclear PLA foci were counted using ImageJ software and plotted with GraphPad Prism.

### Immunoblotting

Immunoblotting of PDOs and cells was done following the procedures previously described by Hogstrom *et al.* (*52*): PDOs were pooled form six wells and incubated for 1.5 hours on ice in Cell Recovery Buffer supplemented with phosphatase inhibitors (Roche, #4906845001). PDOs and breast cancer cells were lysed using radioimmunoprecipitation assay buffer (Boston BioProducts, #BP-115) containing protease and phosphatase inhibitors (Thermo Fisher Scientific, #A32959) and then centrifuged at 13,000 rpm for 10 min at 4°C. Protein concentrations were measured using a Pierce BCA protein assay kit and then boiled in 4× LDS sample buffer (Invitrogen, NP0007). Proteins (20 μg per sample) were separated by electrophoresis on SDS-polyacrylamide gels (Invitrogen, #NP0321BOX or XP04200BOX) and transferred to polyvinylidene difluoride membranes at 25 V overnight at 4°C. The membranes were blocked with 5% nonfat milk in 0.1% Tween 20 in tris-buffered saline for 1 hour and incubated with primary antibodies for 1 hour in room temperature or overnight at 4°C using the following antibodies: FAH (Novus, NBP2-48736), c-MYC/N-MYC (CST, #13987), pRB (CST, #9307), and VCL (CST, #13901). The membranes were then incubated with appropriate horseradish peroxidase–conjugated secondary antibodies (CST, #7074 or Invitrogen, #32430) for 1 hour. Enhanced chemiluminescence was performed using Pierce SuperSignal West Femto Maximum Sensitivity Substrate (Thermo Fisher Scientific, #PI34096) and visualized using Amersham Imager 600. Membranes were stripped for 10 min using 20 mM NaOH and reblotted one time.

### Coimmunoprecipitation

For co-IPs performed on whole-cell extract, cells were lysed in Pierce IP lysis buffer (Thermo Fisher Scientific, #87787) containing complete protease inhibitor cocktail tablet (Thermo Fisher Scientific, #A32959). Cells were then vortexed and centrifuged at 14,000*g* for 10 min at +4°C. One milligram of protein per sample was incubated overnight with 50 μl of Protein A/G beads (Thermo Fisher Scientific, #78609) that were conjugated with 6 μl of CDK9 (CST, #2316S) or 0.5 μl of immunoglobulin G rabbit negative control (CST, #2729). After incubation, beads were washed three times with 0.5% Tween in PBS using magnetic stand, and beads were boiled for 5 min in 2× SDS loading buffer supplemented with 50 mM dithiothreitol. Magnetic stand was used to separate beads, and samples were loaded in 4 to 12% precast bis-tris gels (Invitrogen), and Western blot was performed using anti–V5-tag antibody (Thermo Fisher Scientific, R960-25).

### BioID

MCF7 CTRL-BioID, FAH NLS-BioID, and FAH NES-BioID were treated with 100 nM abemaciclib for 72 hours and 50 μM biotin for 24 hours and were thereafter washed twice with cold PBS. Cells were lysed and processed according to a previously published protocol (*32*). Sonicated lysates were incubated overnight with Dynabeads MyOne Streptavidin C1 (Thermo Fisher Scientific, #65001) on a rotator at +4°C. The following day, the beads were washed five times with 50 mM ammonium bicarbonate. On-bead trypsin digestion and liquid chromatography–tandem mass spectrometry analysis were performed by the Taplin Biological Mass Spectrometry facility (Harvard Medical School, Boston). Enrichment analysis of GO molecular function was performed using the AmiGO gene ontology online database and tool by calculating fold enrichment of GO terms that are overrepresented in the dataset compared to the background set.

### AlphaFold multimer prediction

AlphaFold multimer prediction was performed using the Cosmic^2^ platform (*54*). Human FAH and human CDK9 protein sequences were obtained from UniProt and uploaded to Cosmic^2^. The AlphaFold2 tool was selected, and a task was created using the full_dbs database and a multimer model. Output models were analyzed using PyMOL software.

### Tyrosine tracing and metabolomics

MCF7 EV and FAH cells were grown in tyrosine-free DMEM media supplemented with 5% dialyzed FBS and ^13^C9 ^15^N tyrosine (0.12 g/liter; Cambridge Isotope Laboratories, #CNLM-439-H) for 4 hours, 24 hours, or 7 days. Cells were washed twice with 0.9% NaCl, trypsinized for 3 min, and inactivated with equal volume dialyzed FBS. Cells were pelleted, washed with 0.9% NaCl, and lysed in 300 μl of metabolite extraction buffer [80% methanol, 25 mM ammonium acetate, and 2.5 mM Na-ascorbate prepared in liquid chromatography–mass spectrometry (LC-MS) water, supplemented with isotopically labeled standards (Cambridge Isotope Laboratories, MSK-QReSS1 and MSK-QReSS2)]. Samples were vortexed for 10 s and then centrifuged for 10 min at 18,000*g* to pellet cell debris. Samples were dried under N2 gas flow at 4°C and reconstituted in 20 μl of high-performance liquid chromatography water. Then, 2 μl was injected into a ZIC-pHILIC 150 mm by 2.1 mm (5-μm particle size) column (EMD Millipore) operated on a Vanquish Flex UHPLC Systems (Thermo Fisher Scientific, San Jose, CA, USA).

Chromatographic separation was achieved using the following conditions: the aqueous additive solution consisted of 20 mM ammonium carbonate and 0.1% ammonium hydroxide in water. Mobile phase A was prepared by mixing acetonitrile with the aqueous additive solution (95:5, v/v), while mobile phase B consisted of the aqueous additive solution mixed with acetonitrile (95:5, v/v). Gradient conditions we used were as follows: 0 to 17.7 min: linear gradient from 16.6 to 75% B; 17.5 to 18 min: from 75 to 85% B; 18 to 20 min: hold at 85% B; 20 to 20.5 min: from 85 to 16.6% B; 20.5 to 24 min: hold at 16.6% B at 150 μl/min flow rate. The column oven and autosampler tray were held at 25° and 4°C, respectively. MS data acquisition was performed using a Q Exactive benchtop Orbitrap mass spectrometer equipped with an Ion Max source and a HESI II probe (Thermo Fisher Scientific, San Jose, CA, USA) and was performed in a positive and negative ionization mode in a range of mass/charge ratio (*m/z*) = 70 to 1000, with the resolution set at 70,000, the AGC target at 1 × 10^6^, and the maximum injection time at 20 ms. HESI settings were as follows: sheath gas flow rate: 35; aux gas flow rate: 8; sweep gas flow rate: 1; spray voltage 3.5 (pos); 2.8 (neg); capillary temperature: 320°C; S-lens RF level: 50; aux gas heater temp: 350°C.

### Data analysis for polar metabolites

Relative quantification of polar metabolites was performed with CompoundDiscoverer (CD) 3.4 implementing in-house libraries (470 compounds and 37 internal standards) or Skyline-daily (v25.1.1.271, MacCoss Lab, University of Washington). Molecular formulas with appropriate isotopic labeling were specified, and theoretical *m/z* values for all isotopologues were calculated within Skyline. Extracted ion chromatograms were generated using a 5–parts per million mass tolerance for [M + H] + (positive mode) and [M − H] − (negative mode) adducts. Chromatograms were smoothed using the Savitzky-Golay algorithm, and all peaks were manually inspected to ensure accurate integration, consistent retention times, and isotopologue coelution. Pooled samples and fractional dilutions were prepared as quality controls and injected at the beginning and end of each run. In addition, pooled samples were interspersed throughout the run to control for technical drift in signal quality as well as to serve to assess the coefficient of variability (CV) for each metabolite. Post-CD, data were further consolidated with an in-house R script: https://doi.org/10.5281/zenodo.20297592. This script (i) extracts and combines the peak areas from CD output.csvs, (ii) calculates and normalizes to an averaged factor from all mean-centered chromatographic peak areas of isotopically labeled amino acid internal standards within each sample, and (iii) reports on low-quality metabolites based on user inputted cutoffs calculated from pool reinjections and pool dilutions. In this study, the linear correlation between the dilution factor and the peak area cutoffs is set to RSQ > 0.95 and the CV < 30%.

### Data analysis for isotopologue analysis

Natural isotope abundance correction was not applied, as the mass shifts associated with the fully labeled tracers (e.g., +4 Da for fumarate and +10 Da for [U-^13^C_9_,^15^N]-tyrosine) were sufficient to prevent overlap with naturally occurring isotopologues. A low concentration of [U-^13^C_4_]-fumaric acid (99%) from the MSK-QReSS2 standard mix was added during extraction and was consistent across all samples. Fumarate signal was uniform across conditions, suggesting that it primarily reflects the external spike-in, with no detectable contribution from endogenous labeled metabolism. Peak areas were used to calculate isotopologue distributions and percent labeling. For quantitative comparisons, peak areas were normalized using an internal normalization factor derived from QC-passing metabolites per sample (*R*^2^ > 0.95; CV < 30%), consistent with targeted metabolomic analysis.

### Reverse transcription quantitative PCR

Three replicates of patient #26 EV and FAH PDOs treated with 300 nM abemaciclib were lysed in guanidine-isothiocyanate buffer, and RNA was isolated using the RNeasy Fibrous Tissue Mini Kit (QIAGEN, #74704). One microgram of RNA was reverse transcribed with the High-capacity cDNA Reverse Transcription Kit (Thermo Fisher Scientific, #4368814), and reverse transcription quantitative PCR was performed using PowerTrack SYBR Green Master Mix for quantitative PCR (Thermo Fisher Scientific, #A46109) and primers for human *HPRT1*, human *FAH*, human *MYCC*, and human *BMYB*. The following primers were used: *MYCC* GAGAAGCTGGCCTCCTACC (forward) and CTGTGAGGAGGTTTGCTGTG (reverse), *BMYB* TACAGCTCAACTCCCTGCC (forward) and TGGAGTGGAGTGGTGTTCTC (reverse), *FAH* TCAGCATCATCAAGCACCTC (forward) and GTCTCCTATGGTGGCTGGAA (reverse), and *HPRT1* GTAATTGGTGGAGATGATCTCTCAACT (forward) and TGTTTTGCCAGTGTCAATTATATCTTC (reverse). For each analysis, three biological replicates and three technical replicates were used. Real-time PCR results were calculated according to the following protocol: Relative expression level = 2-ΔCt, where ΔCt = Ct (gene of interest) − Ct (housekeeping gene). Human *HPRT1* was used as the housekeeping gene.

### RNA sequencing

Three replicates of MCF7 EV and FAH cells were treated with 200 nM abemaciclib and lysed in guanidine-isothiocyanate buffer, and RNA was isolated with the RNeasy Fibrous Tissue Mini Kit as above. Before library prep, the RNA quality was assessed with the Bioanalyzer (Agilent). The mRNA libraries were generated by Novogene, and bulk RNA-seq was done using the Illumina NovaSeq platform. Raw sequencing reads were aligned to the Ensembl v104 human reference transcriptome with salmon v1.4.0 using options “--seqBias --useVBOpt --gcBias --posBias --numBootstraps 30 --validateMappings.” Length-scaled transcripts per million were acquired using the tximport v1.18.0 function, and log_2_ fold changes and false discovery rates (FDRs) were determined by DESeq2 v1.30.1 in R. Signed −log_10_ FDRs from DESeq2 analyses were used to rank genes for GSEA via fgsea v1.16.0, filtering out genes with an FDR > 0.2. GO Biological Processes (GO.db v3.12.1) pathway-to-gene mappings were used, with Ensembl gene IDs matched to corresponding Entrez IDs via biomaRt v2.46.3.

Four replicates of T47D gAAVS and gFAH #3 cells were treated with 500 nM abemaciclib for 3 days. Cells were trypsinized for 2 min, washed with PBS, and resuspended in Zymo DNA/RNA Shield (Thermo Fisher Scientific, 50-444-614). RNA isolation and the mRNA libraries were generated by Plasmidsaurus, and bulk RNA-seq was performed using the Illumina NovaSeq platform. Quality of the fastq files was assessed using FastQC v0.12.1. Reads were then quality filtered using fastp v0.24.0 with poly-X tail trimming, 3′ quality-based tail trimming, a minimum Phred quality score of 15, and a minimum length requirement of 50 bp. Quality-filtered reads were aligned to the reference genome using STAR aligner v2.7.11 with noncanonical splice junction removal and output of unmapped reads, followed by coordinate sorting using samtools v1.22.1. PCR and optical duplicates were removed using UMI-based deduplication with UMIcollapse v1.1.0. Alignment quality metrics, strand specificity, and read distribution across genomic features were assessed using RSeQC v5.0.4 and Qualimap v2.3, with results aggregated into a comprehensive quality control report using MultiQC v1.32. Gene-level expression quantification was performed using featureCounts (subread package v2.1.1) with strand-specific counting, multimapping read fractional assignment, exons, and 3′ untranslated region as the feature identifiers and grouped by gene_id. Final gene counts were annotated with gene biotype and other metadata extracted from the reference GTF file. Sample-sample correlations for sample-sample heatmap and principal components analysis were calculated on normalized counts (trimmed mean of *M* values) using Pearson correlation. Differential expression was done with edgeR v4.0.16 using the standard practice including filtering for low-expressed genes with edgeR::filterByExprwith default values. Gene set enrichment analysis was performed with gseapy v0.12 using the Hallmark gene set, MSigDB C2, and Shepard BMYB targets and Yu MYC targets up through the MSigDB C2 gene sets (*55**–**58*).

### RNA-seq of publicly available datasets

RNA-seq data for breast invasive carcinoma (BRCA) were obtained from TCGA via the Genomic Data Commons (GDC) Data Portal. The RNA-seq data and the clinical metadata were downloaded separately by adding the data to the GDC cart, exporting the Manifest file, and then using the GDC Data Transfer Tool to complete the download. The dataset comprised 1211 samples, including 1097 tumor samples and 114 adjacent normal samples. Gene expression data, quantified as RNA-seq by expectation maximization (RSEM) values, were downloaded as raw read counts and normalized to log_2_(RSEM + 1) for downstream analysis. Clinical metadata, including sample type (tumor or normal) and molecular subtype annotations (e.g., HR+ and HER2), were retrieved from the TCGA Pan-Cancer Atlas (*23*). Data preprocessing was performed using Python (v3.13) with the pandas (v2.2.2) and NumPy (v1.26.4) libraries to ensure consistent gene identifiers (Ensembl IDs) and remove low-quality samples with missing expression data.

The tyrosine catabolism pathway was analyzed by focusing on five key genes: *TAT* (ENSG00000198650), *HPD* (ENSG00000158104), homogentisate 1,2-dioxygenase (*HGD*; ENSG00000113924), glutathione *S*-transferase zeta 1 (*GSTZ1*; ENSG00000100577), and *FAH* (ENSG00000103876). Mean log_2_(RSEM + 1) expression values for each gene were calculated separately for tumor and normal samples using pandas. Differential expression was assessed by comparing mean expression between tumor and normal groups, with statistical significance determined using a two-sample *t* test (SciPy v1.13.1, *P* < 0.05). Results were visualized using barplots and boxplots generated with seaborn (v0.13.2) and matplotlib (v3.8.4), comparing expression levels across sample types.

To compare the enrichment of the tyrosine metabolism pathway (KEGG, hsa00350) with other amino acid metabolism pathways, GSEA was implemented in Python using a custom pipeline. Gene sets for amino acid metabolism pathways, including cysteine and methionine metabolism (hsa00270), alanine, aspartate, and glutamate metabolism (hsa00250), glycine, serine, and threonine metabolism (hsa00260), arginine and proline metabolism (hsa00330), phenylalanine metabolism (hsa00360), tryptophan metabolism (hsa00380), valine, leucine, and isoleucine degradation (hsa00280), histidine metabolism (hsa00340), and lysine degradation (hsa00310), were obtained from the KEGG Pathway Database. For each gene, the log_2_ fold change of expression between tumor and normal samples was calculated to create a ranked gene list. Enrichment scores were computed by evaluating the running sum of ranked genes within each pathway, with significance assessed using 1000 permutations to generate null distributions. Normalized enrichment scores, *P* values, and FDR *q*-values were calculated, with pathways having *P* < 0.05 and FDR *q* < 0.1 considered significantly enriched. The analysis was performed using NumPy and pandas, with results visualized as a barplot of normalized enrichment scores using seaborn, highlighting the relative enrichment of tyrosine metabolism among other pathways. All statistical analyses were conducted in Python. For the tyrosine catabolism gene analysis, mean expression differences were tested for significance using two-sample *t* tests, with *P* values adjusted for multiple comparisons using the Bonferroni correction. For GSEA, statistical significance was determined on the basis of permutation-based *P* values and FDR *q*-values computed in the Python pipeline. Visualizations, including barplots, boxplots, heatmaps, and violin plots, were generated to illustrate gene expression patterns and pathway enrichment.

### Statistical analysis

Statistical analysis was performed using Prism GraphPad software for all in vitro and ex vivo experiments. All experiments with MCF7 cells were repeated at least twice with six to eight technical replicates. Experiments with PDOs were performed as two to four biological replicates, and 10 to 20 technical replicates were included for each individual experiment. Data are presented as mean ± SD unless otherwise stated in the figure legend. Student’s unpaired *t* test, two-sample *t* test, one-way analysis of variance (ANOVA) followed by Dunnetts or Dunn’s multiple corrections test, Welch *t* test, or Mann-Whitney test was used to assess significance. For nuclear FAH in patient tumors, R was used for statistical analysis. Patient datasets were first tested for normality using the Shapiro-Wilk test. When found to be nonnormal, pairwise comparison of data was carried out using the Wilcoxon rank sum test, using the Holm-Bonferroni method to correct for a family-wise error.
