# Supplementary material for: A noncanonical function of the tyrosine degradation enzyme FAH drives CDK4/6 inhibitor resistance in breast cancer
Source: Sci Adv. 2026 Sep 30;12(40):eaef1145. doi: 10.1126/sciadv.aef1145 (PMC13626039; doi:10.1126/sciadv.aef1145)
Supplement: Supplementary file 1 [file sciadv.aef1145_sm.pdf]

Science Advances
AAAS

Supplementary Materials for

A noncanonical function of the tyrosine degradation enzyme FAH drives CDK4/6 inhibitor resistance in breast cancer

Jenny Högström *et al.*

Corresponding author: Naama Kanarek, naama.kanarek@childrens.harvard.edu;
Taru Muranen, tmuranen@bidmc.harvard.edu

Sci. Adv. **12**, eaef1145 (2026)
DOI: 10.1126/sciadv.aef1145

The PDF file includes:

Figs. S1 to S6
Legends for data S1 to S4

Other Supplementary Material for this manuscript includes the following:

Data S1 to S4

Figure S1

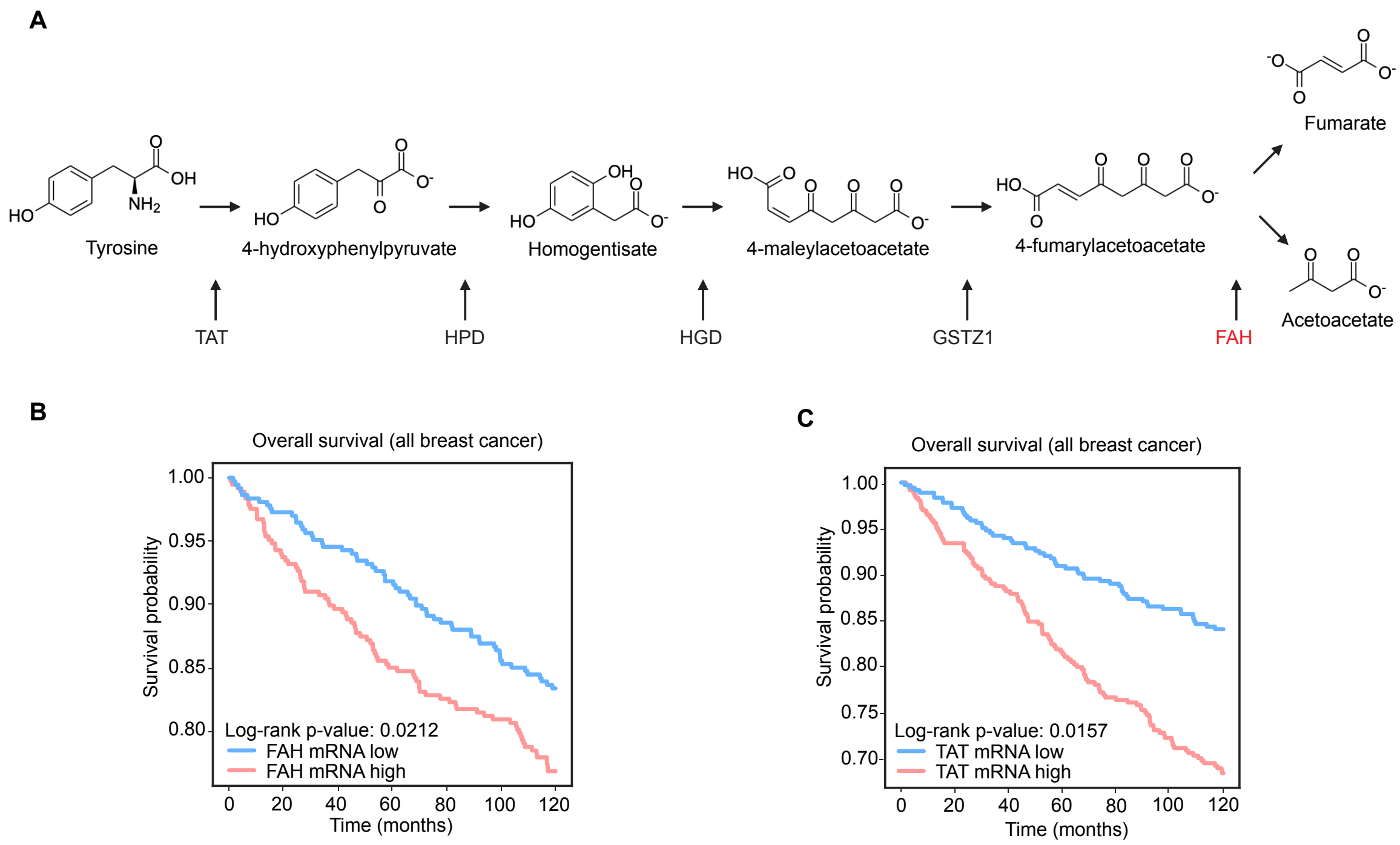

Fig. S1. FAH is enriched in breast cancer and correlates with poor overall survival. A) Illustration of the canonical tyrosine degradation pathway. B-C) Kaplan-Meyer plot showing overall survival of FAH high vs low mRNA (B) and TAT high vs low mRNA (C) in all breast cancer cases from TCGA. A tertile-based cutoff was used to determine FAH high and low mRNA. The TCGA Pan-cancer Atlas data set was used for the analysis. Mantel-Cox log rank test was used to assess significance.

Figure S2

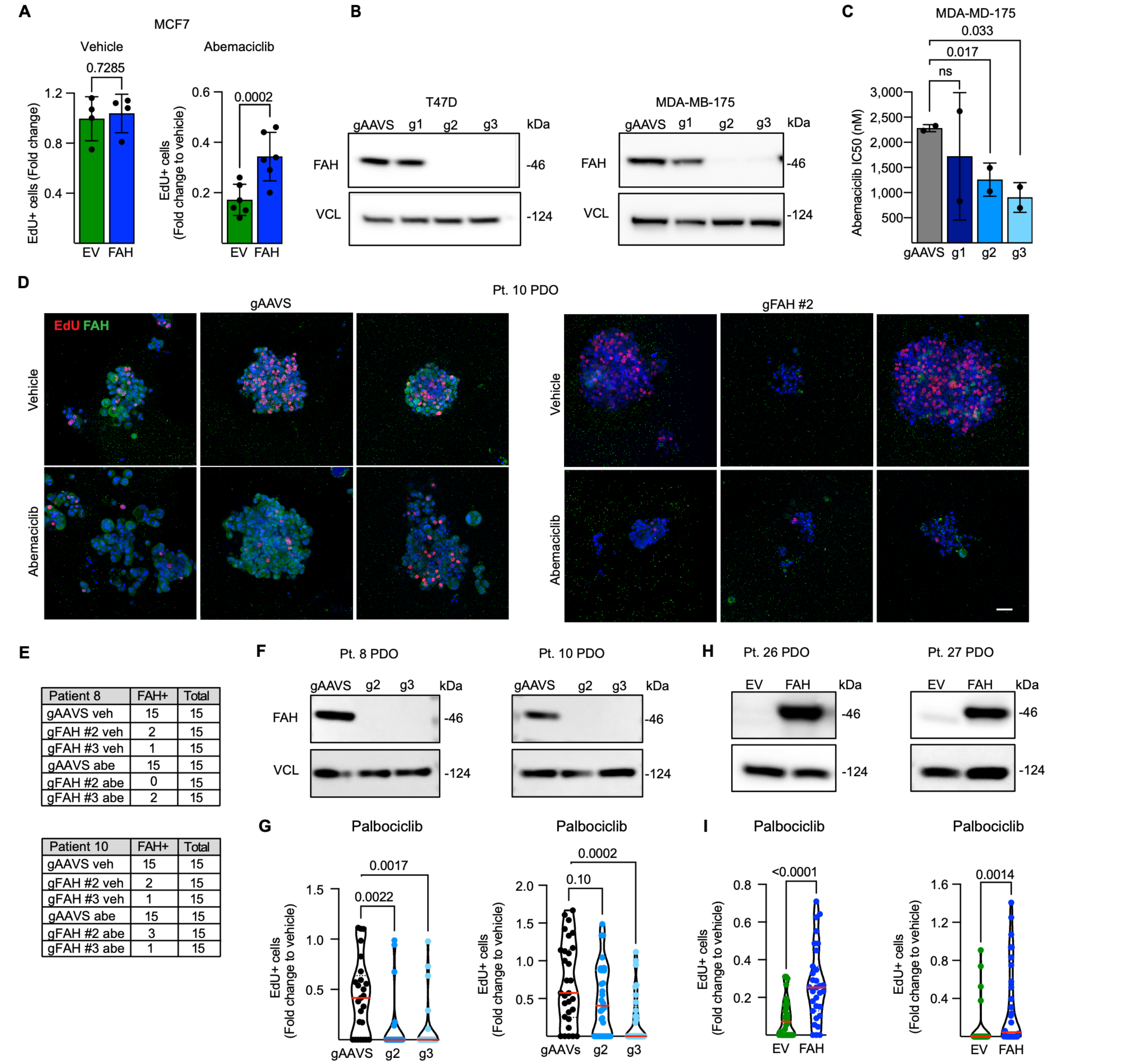

Patient 8	FAH+	Total
gAAVS veh	15	15
gFAH #2 veh	2	15
gFAH #3 veh	1	15
gAAVS abe	15	15
gFAH #2 abe	0	15
gFAH #3 abe	2	15

Patient 10	FAH+	Total
gAAVS veh	15	15
gFAH #2 veh	2	15
gFAH #3 veh	1	15
gAAVS abe	15	15
gFAH #2 abe	3	15
gFAH #3 abe	1	15

Fig. S2. FAH modulation affects resistance to CDK4/6-inhibitors. A) Quantification of EdU+ cells in empty vector (EV) control and FAH overexpressing MCF7 cells treated with vehicle (A) or 200 nM abemaciclib for 72h. Proliferation was assessed by pulsing cells for 2h with EdU. B) FAH and vinculin (VCL) protein in MDA-MB-175, and T47D gAAVS control and gFAH KO cells. C) IC50 value in MDA-MB-175 CTRL and gFAH cells treated with increasing concentrations of Abemaciclib for 72h. PrestoBlue was used to assess cell viability. Two independent experiments were used to assess significance. D-E) Representative images of patient #10 PDOs (D) and scoring of FAH positive PDOs (E) in patient #8 and #10 PDOs treated with 300 nM abemaciclib for 7 days. F) FAH and VCL protein in patient #8 and #10 gAAV and gFAH PDOs. G) Ratio of EdU+ cells in patients #8 and #10 PDOs treated with 300 nM abemaciclib for 7 days. H) Western blot of FAH and VCL protein in patients #26 and #27 EV- and FAH-overexpressing PDOs. I) Ratio of EdU+ cells in patient #26 and EV- and FAH-overexpressing PDOs treated with 300 nM palbociclib for 7 days. Proliferation was assessed by pulsing PDOs with EdU for 4h and quantifying the ratio of EdU+ cells per total (DAPI+) number of cells in 15-20 PDOs in each independent experiment. Biological replicates: n = 6 (A), n = 2 (C, G, I). Error bars indicate mean+SD, red line indicates median. Student's unpaired t-test (A, I), one-way ANOVA with Dunnett's test (G), and Welch t-test (C) was used to assess significance. Scalebar: 40 μm.

Figure S3

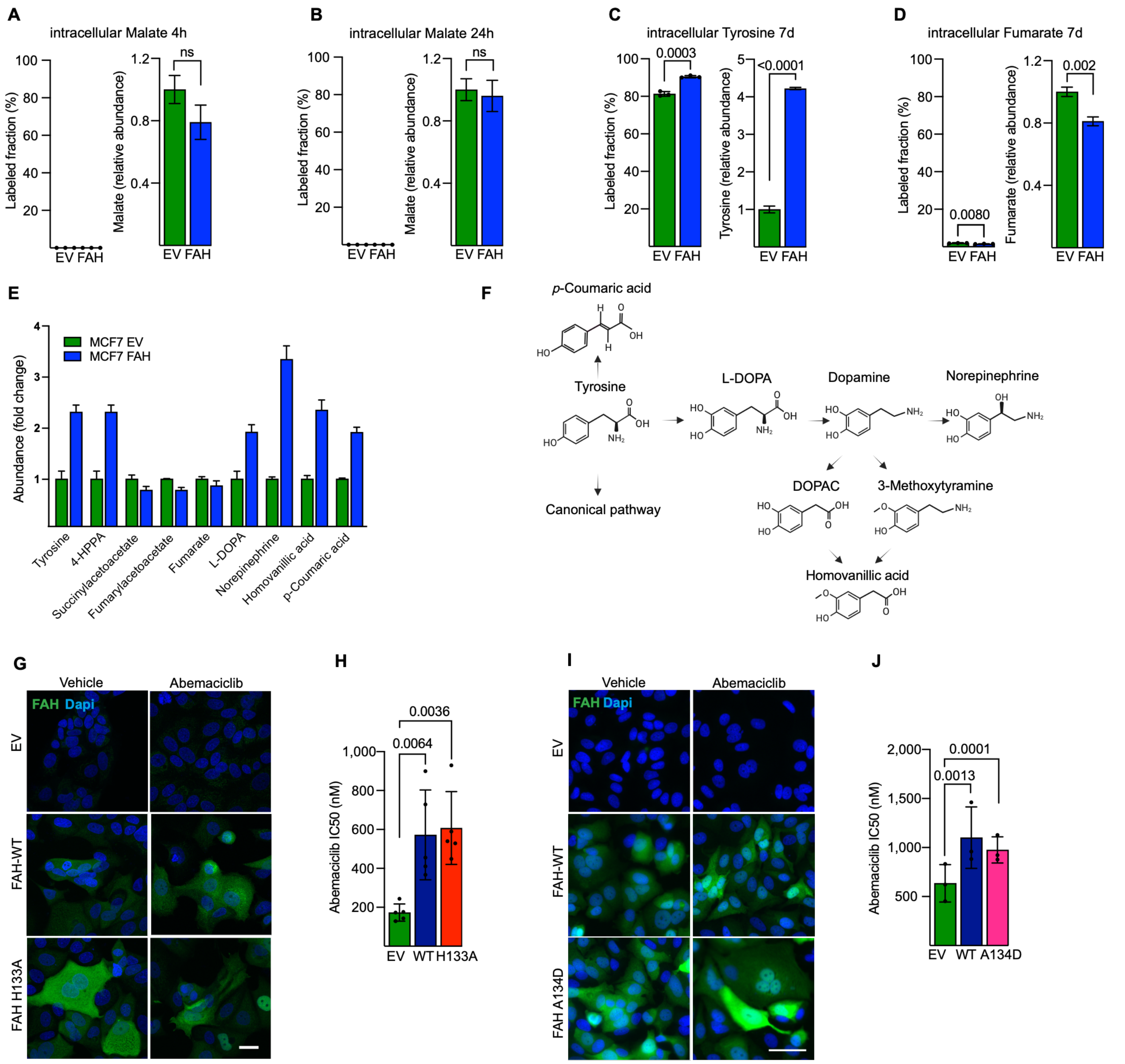

Fig. S3. Active tyrosine degradation pathway is not required for FAH-mediated resistance to CDK4/6-inhibitors. A-B) Percentage of ^{13}C labeling and abundance of intracellular malate at 4h (A) and 24h (B). C-D) Percentage of ^{13}C labeling and abundance of intracellular tyrosine (C) and intracellular fumarate (D) at 7 days. Tyrosine labeling was performed by growing MCF7 empty vector (EV) control and FAH cells in media supplemented with 5% dialyzed FBS and 0.12 g/L $^{13}C9$ ^{15}N Tyrosine for 4h (A), 24h (B) or 7 days (C, D). E) Abundance of tyrosine catabolism pathway metabolites in MCF7 EV and FAH overexpressing cells grown in media supplemented with 5% dialyzed FBS for 7 days. F) Illustration of non-canonical tyrosine catabolism pathway. Created in Biorender. Muranen, T. (2026) https://BioRender.com/chqhmbk. G) Representative images of FAH staining in MCF7 EV, FAH WT and FAH H133A cells treated with 200 nM abemaciclib for 72h. H) Abemaciclib IC50 in MCF7 EV, FAH WT and FAH H133A cells treated with increasing doses of abemaciclib for 72h. I) Representative images of FAH staining in MCF7 EV, FAH WT and FAH A134D cells treated with 200 nM abemaciclib for 72h. J) Abemaciclib IC50 in MCF7 EV, FAH WT and FAH A134D cells treated with increasing doses of abemaciclib. Biological replicates: n= 5 (H), n= 3 (J). Error bars indicate mean+SD. Student's unpaired t-test (A-D), one-way ANOVA with Dunnett's test (H), and Welch t-test (J) was used to assess significance. Scalebar: 10 μm (G), 40 μm (I).

Figure S4.

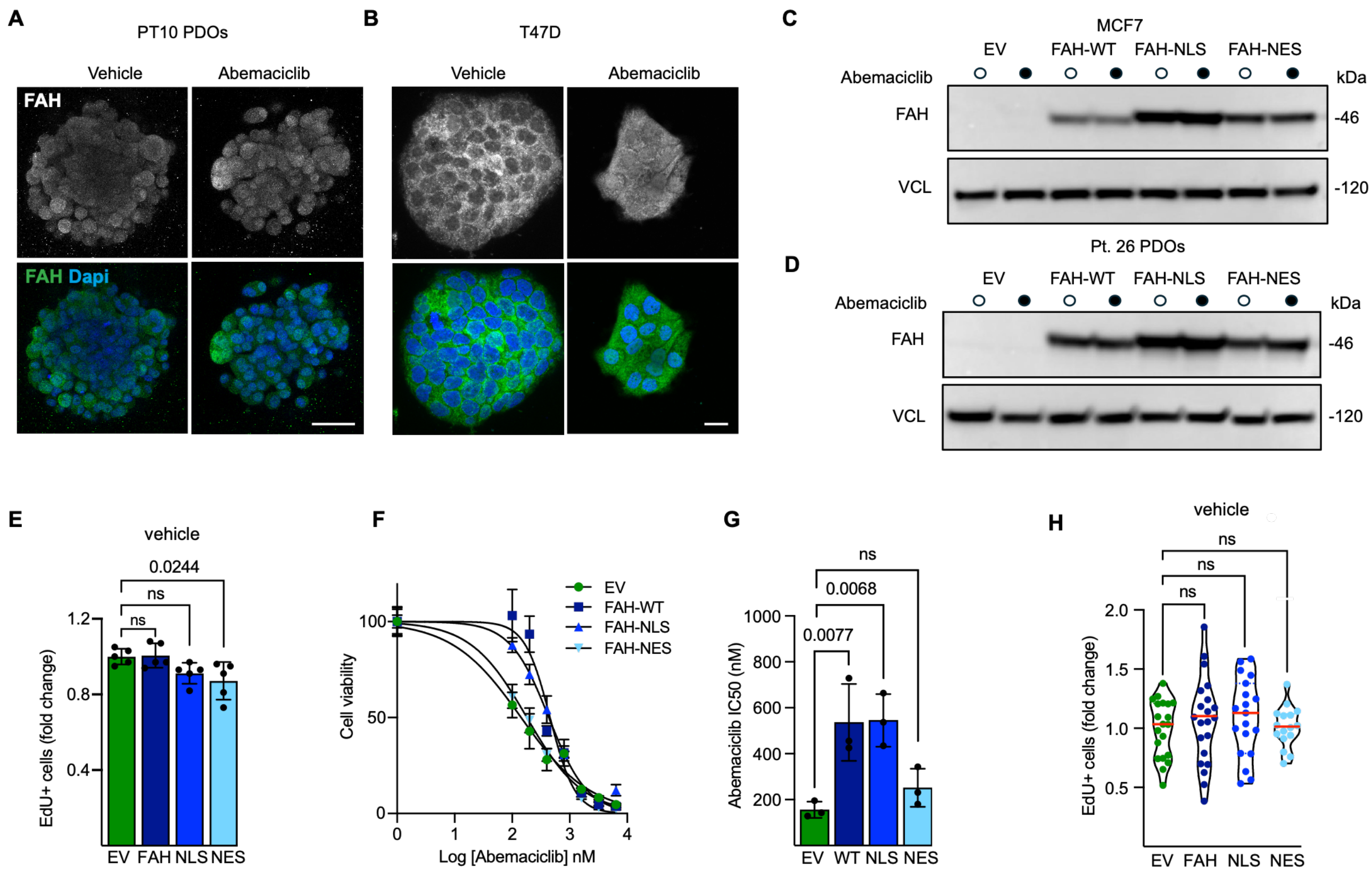

Fig. S4. FAH is expressed in the nucleus after CDK4/6-inhibitors therapy. A) FAH and DAPI staining of patient #10 PDOs treated with 300 nM abemaciclib for 7 days. B) FAH and DAPI staining of T47D cells treated with 500 nM abemaciclib for 72. C-D) FAH and vinculin (VCL) protein in MCF7 cells (C) and patient #26 (D) with different FAH constructs expressed (EV control, WT, NLS and NES). E) Quantification of EdU+ cells of vehicle treated MCF7 EV, FAH-WT, FAH-NLS and FAH-NES cells. Proliferation was assessed by pulsing MCF7 cells with EdU for 2h. F-G) Abemaciclib IC50 curve (F) and IC50 values (G) in MCF7 EV, FAH-WT, FAH-NLS and FAH-NES cells treated with increasing doses of abemaciclib for 72h. H) Quantification of EdU+ cells of vehicle treated patient #26 EV, FAH-WT, FAH-NLS and FAH-NES PDOs. Proliferation was assessed by pulsing PDOs with EdU for 4h and quantifying the ratio of EdU+ cells per total (DAPI+) number of cells in 15-20 PDOs from each independent experiments. Biological replicates: n=3 (G), n=5 (E), n=2 (H). Error bars indicate mean+SD, red line indicates median. One-way ANOVA with Dunnett's test (E, G, H) was used to assess significance. Scalebar: 40 μm (A), 10 μm (B).

Figure S5.

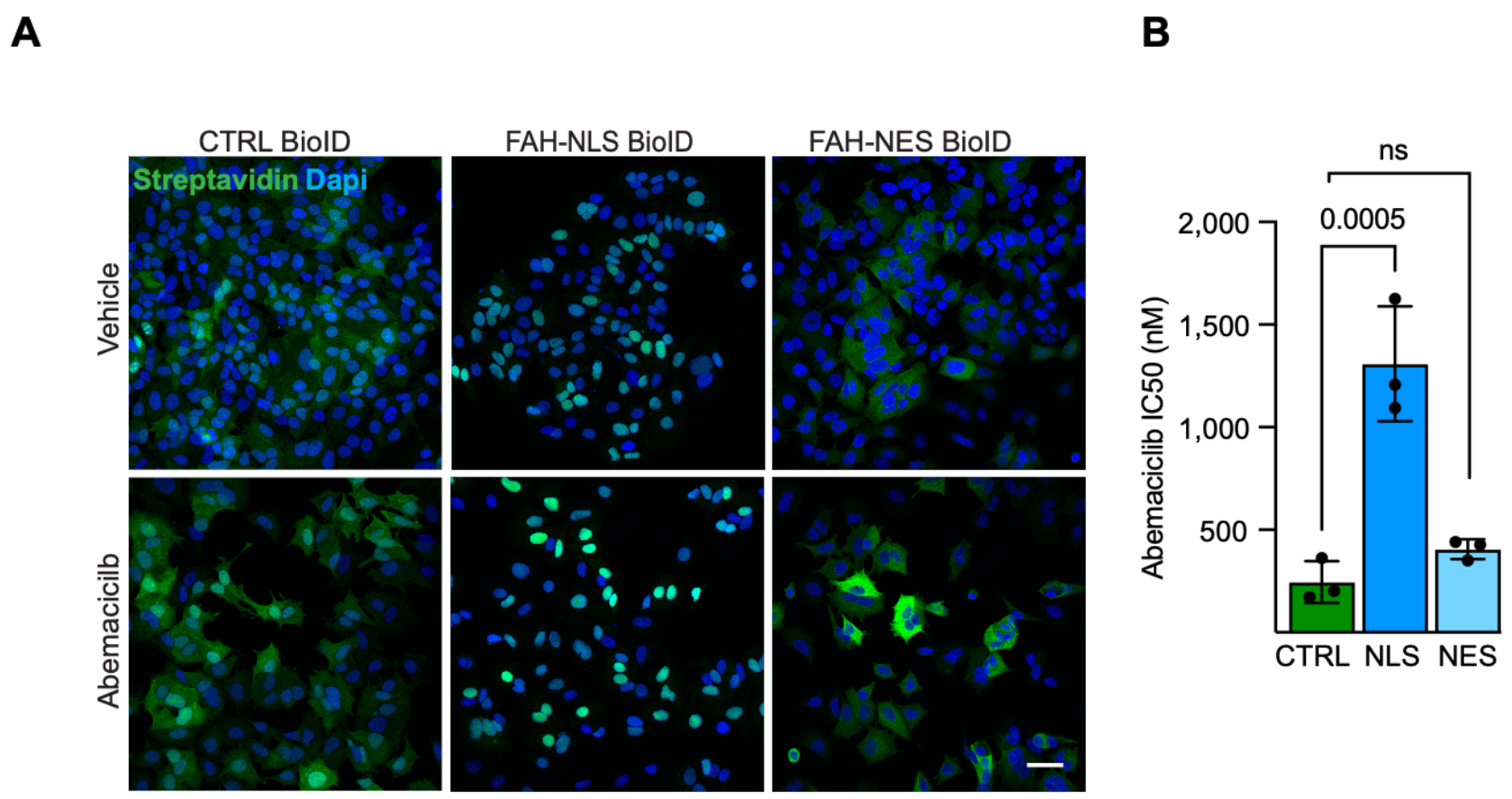

C

Gene symbol	CTRL Vehicle	NLS Vehicle	NES Vehicle
FAH	0	33	35
CHD4	4	18	0
BRIP1	1	15	0
PAPOLA	3	14	0
KMT2A	2	13	0
KDM3B	2	12	0
WDR50	2	12	0
CMTR1	1	12	0
STAG2	2	11	0
SBNO1	1	9	0

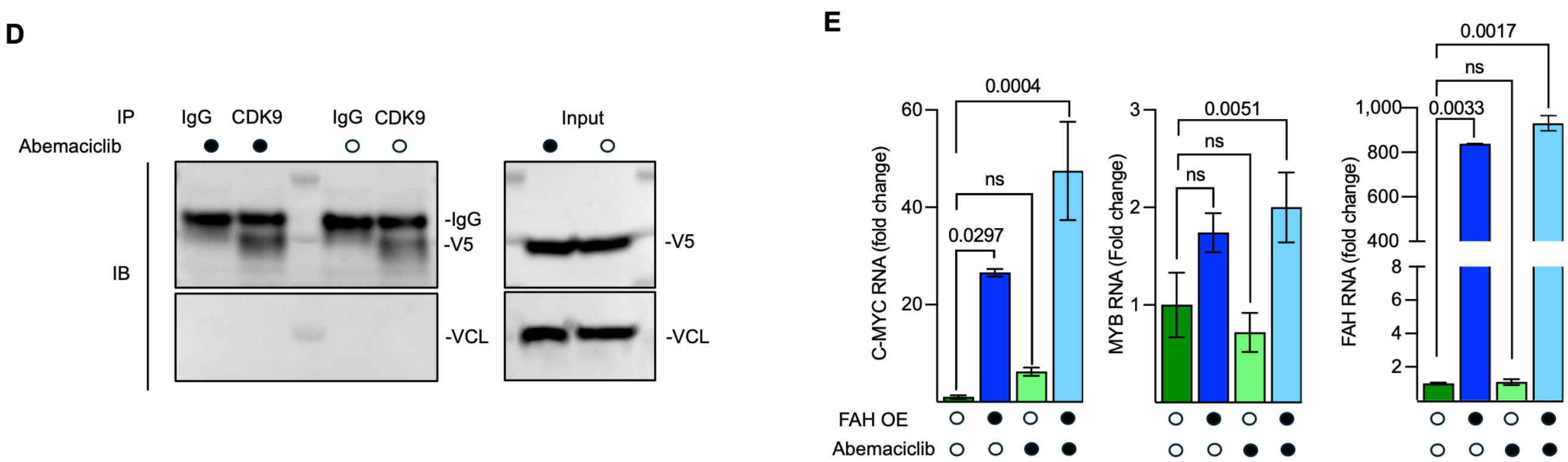

Figure S5. FAH interacts with CDK9. Representative images of Streptavidin and DAPI staining in MCF7 CTRL BioID, FAH-NLS BioID and FAH-NES BioID cells treated with 200 nM Abemaciclib for 72h and pulsed with 50 μM biotin for 24h. B) IC50 value of MCF7 CTRL, FAH-NLS and FAH-NES BioID cells treated with increasing doses of Abemaciclib for 72h. C) Top ten proteins detected in MCF7 CTRL, FAH-NLS and FAH-NES BioID cells treated with vehicle for 72h. Table shows average peptide counts from triplicate samples analyzed by mass spectrometry. CTRL BioID and FAH-NES BioID peptide counts are shown as controls. D) V5-tag immunoblot of IgG control and CDK9 co-immunoprecipitation of MCF7 FAH cells treated with vehicle or 200 nM Abemaciclib for 72h. Vinculin (VCL) was used as loading control. E) c-MYC, MYB and FAH RT-qPCR in vehicle or abemaciclib treated patient #26 empty vector (EV) control and FAH PDOs. Biological replicates: n= 3 (B, E). One-way ANOVA with Dunnett's test (B, E) was used to assess significance. Scalebar: 40 μm

Figure S6

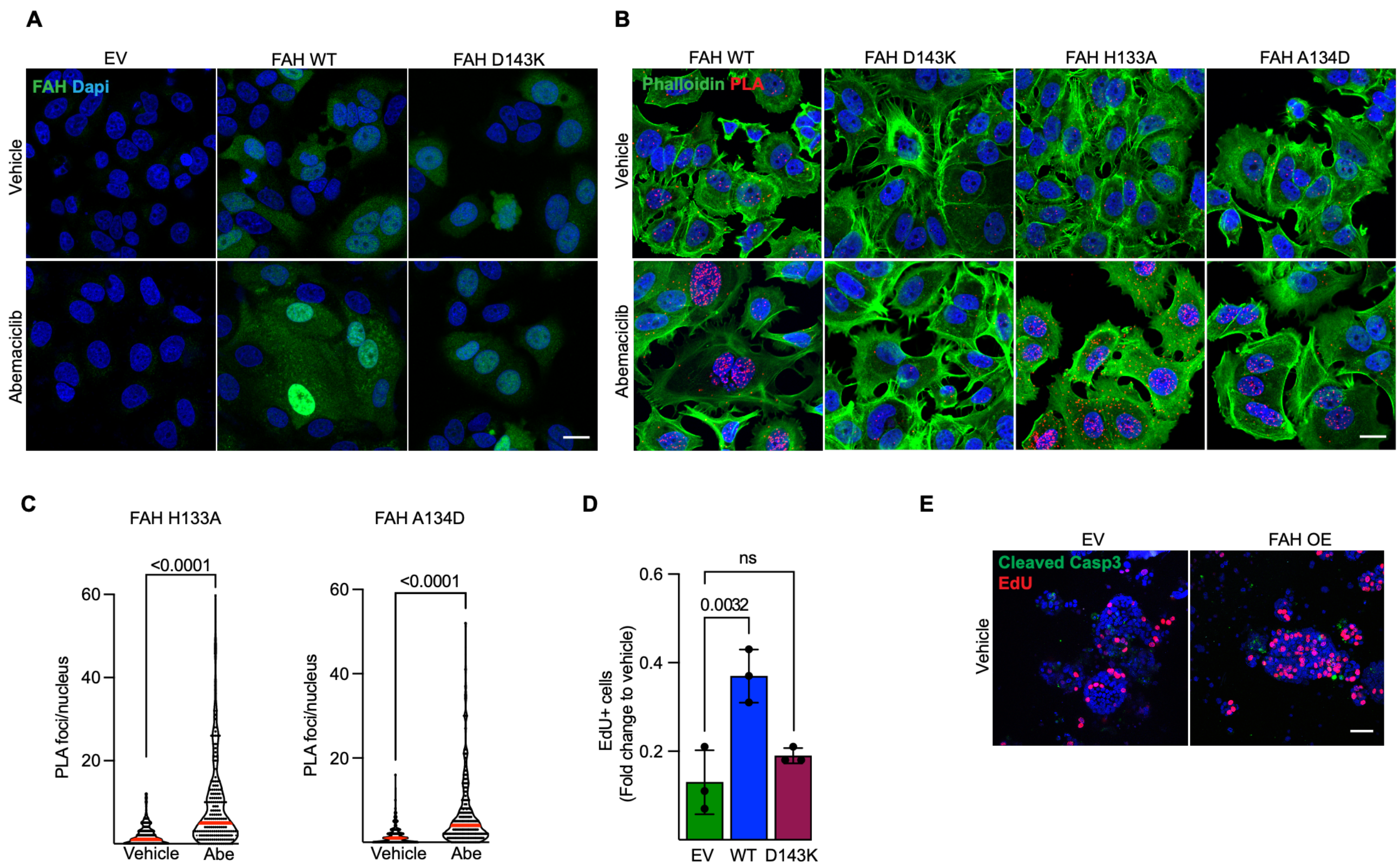

Figure S6. FAH interacts with CDK9 to drive resistance to CDK4/6-inhibitors. A) Representative images of FAH and DAPI staining in MCF7 empty vector (EV) control, FAH WT and FAH D143K cells treated with 200 nM abemaciclib for 72h. B) Representative images of proximity ligase assay (PLA) foci between FAH and CDK9 and phalloidin staining (B) and quantification (C) of nuclear PLA foci between CDK9 and FAH in FAH H133A and FAH A134D cells. D) Quantification of EdU+ cells in MCF7 EV, FAH WT and FAH D143K cells treated with 200 nM abemaciclib for 72h. Proliferation was assessed by pulsing cells for 2h with EdU. E) Representative confocal images of Cleaved Caspase 3 and EdU staining in vehicle treated patient #26 EV and FAH PDOs. Biological replicates: n=2 (C), n=3 (D). Error bars indicate mean+SD, red line indicates median. Mann-Whitney (C) test or one-way ANOVA with Dunnett's test (D) was used to assess significance. Scalebar: 10 μm (A, B) 40 μm (E).

Supplementary data files:

Data S1. This file contains data regarding the patients used in the tissue microarray, patient-derived organoid cultures and pre and post CDK4/6-inhibitor tumors

Data S2. This file contains metabolite peak values used to assess tyrosine tracing and total metabolite abundance

Data S3. This file contains analyzed RNA-sequencing data

Data S4. This file contains raw peptide counts from proteomics and analyzed data
